# TBS-pyrrole as an “universal” reference to quantify artemisinin and structurally-diverse natural products in plants extracts by NMR

**DOI:** 10.3389/fpls.2023.1255512

**Published:** 2023-09-29

**Authors:** Ana L. García-García, Dácil Hernández, Álvaro Santana-Mayor, David Jiménez-Arias, Alicia Boto

**Affiliations:** ^1^ Grupo de Síntesis de Fármacos y Compuestos Bioactivos, Instituto de Productos Naturales y Agrobiología del Consejo Superior de Investigaciones Científicas (CSIC), La Laguna, Spain; ^2^ Programa de Doctorado de Química e Ingeniería Química, Universidad de La Laguna, San Cristóbal de La Laguna, Spain; ^3^ Fundación Canaria General de la Universidad de La Laguna, Edificio Servicios Generales de Apoyo a la Investigación (SEGAI), San Cristóbal de La Laguna, Spain; ^4^ Isoplexis-Centro de Agricultura Sustentável e Tecnologia Alimentar, Universidade da Madeira, Funchal, Portugal; ^5^ Instituto Canario de Investigaciones Agrarias, La Laguna, Spain

**Keywords:** NMR analysis, NMR quantification, NMR internal standard, HPLC, artemisinin, natural products, plant extraction

## Abstract

The commercial production of artemisinin and other valuable bioactive natural products depends on their plant sources, which may provide variable amounts of the compound depending on plant variety, the period of the year, abiotic stress and other factors. Therefore, it requires a method for large-scale, low-cost natural product quantification. The standard HPLC and UHPLC methods are accurate but the analysis are costly and require different optimization for structurally-diverse products. An alternative method using NMR with TBS-pyrrole as a novel “universal” reference affords a simple, fast method to quantify many different products. The method is shown with antimalarial artemisinin, whose yield using conventional and novel extraction procedures was determined by standard UHPLC-MS procedures and by our NMR protocol, with similar quantification results. The novel reference compound does not interfere with artemisinin or extract signals, only needs a small amount of the extract, is accurate and operationally simple, and a large volume of samples can be processed in little time. Moreover, bioactive terpenes, steroids, alkaloids, aromatic compounds, and quinones, among others, were quantified in a model vegetal extract with this “universal” reference with excellent accuracy.

## 1 Introduction

Many active principles used as drugs, food additives and/or fine chemicals are plant natural products or semisynthetic derivatives, such as the antimalarial artemisinin (Klayman, 1985; Hien et al., 1993; O’Neill and Posner, 2004; Li and Zhou, 2010), or quinine, which not only was the first antimalarial drug (Achan et al., 2011), but is also used as food additive to give a bitter flavouring in tonic water and other beverages (Tong et al., 2009), and also as a chiral ligand and organocatalyst (Li et al., 2022). Although different synthetic routes to such compounds have been developed, they often have limitations for large-scale production, and therefore, in many cases commercial availability is based on plant extraction (Sirirungruang et al., 2022). In the last years, attempts to increase their production using plant biotechnology, elicitors, and modified culture conditions have attracted much interest (García-García et al., 2020; Khare et al., 2020; Abdulhafiz et al., 2022; Das et al., 2022; Graziani et al., 2022; Sirirungruang et al., 2022). However, plant extraction has drawbacks. For instance, the need to quantify the amount of the active principle in the extracts, in order to homogenize the formulations.

The commercial production of the potent antimalarial and antimicrobial artemisinin (ART) by *Artemisia annua* illustrates this point. The natural source provides variable amounts of the compound depending on plant variety, the period of the year, abiotic stress and other factors (Tellez et al., 1999; Ferreira et al., 2005; Jha et al., 2011; Nganthoi and Sanatombi, 2019). The modification of the extraction conditions can also provide a multi-fold increase of artemisinin isolation (Nahar et al., 2020). In any case, commercial production requires a method for large-scale, low-cost artemisinin quantification in samples. One of the best methodologies to achieve this goal is High Performance Liquid Chromatography (HPLC) and related Ultra-High Performance Liquid Chromatography (UHPLC) (Zhao and Zeng, 1985; Jessing et al., 2011; Nahar et al., 2020). This chromatographic methodology is accurate and reliable, but the analysis are costly, due to the requirement of ultra-pure solvents and appropriate columns, and time costs. Besides, artemisinin lacks chromophores and requires derivatization prior to UV detection (Kontogianni et al., 2020). Therefore, in order to process a large amount of samples, the artemisinin producer will invest much time and have more costs.

In case that the producer needs to quantify structurally-diverse natural products, different HPLC or UHPLC conditions, including columns and solvents, should be optimized. In addition, different internal standards (one per analyte) would be required, which may not be commercially available. We reasoned that an alternative protocol based on Nuclear Magnetic Resonance (NMR) with an “universal” reference could simplify the analysis and would be also quite accurate (Castilho et al., 2008; Kontogianni et al., 2020). Moreover, although the NMR equipment is more expensive than the HPLC/UHPLC equipment, many public and private entities offer NMR services at a very reasonable price, which is usually much lower than the price of an HPLC analysis. This is due to its operational simplicity, which saves personnel and sample preparation costs, with fast optimization of acquisition parameters and very fast processing of the samples.

Similarly to HPLC, in quantitative NMR (qNMR) an internal standard is mixed with the sample containing the desired natural product/s. By measuring the areas of selected signals of the reference substance (whose amount is known) and the target product, it is possible to work out the relationship between the amount of both compounds. However, the NMR protocol is much simpler than HPLC, consisting of mixing solutions of extract and reference in a NMR tube and recording the spectrum. Any amount of the standard and extract would do (in a certain range), but to be suitable, the reference signals should not overlap with those of the target product or the extract. If the separation is not good, then HPLC techniques or an NMR/HPLC combination should be used (Soininen et al., 2014). Despite this, as will be seen in the Article, many bioactive natural products possess some differentiated signal/s and the NMR quantification can be applied.

Some authors have compared NMR and other techniques such as HPLC, LC or TLC for the quantification of artemisinin. For instance, Castilho et al. (2008) used t-butanol (t-BuOH) as an internal standard in artemisinin extracts and compared the ^1^HNMR results with the HPLC-refractive index (HPLC-RI) ones. The first method gave a linear response for artemisinin, using the concentration range 9.85–97.99mM (R^2 =^ 0.9968). For two similar sample concentrations, the calculated artemisinin yields using NMR and HPLC-RI were 0.77% versus 0.72% and 0.86% versus 0.71%. These differences could be due to the standard choice since t-BuOH (δ_H_ 1.26) usually overlaps with some extract signals at high yields.

Liu et al. (2010) improved the method by replacing t-BuOH by maleic acid as a standard. Maleic acid presents a unique sharp signal in d-methanol solution at δ_H_ 6.28, corresponding to two equivalent protons. This signal did not overlap with that of artemisinin at δ_H_ 6.02, or other extract signals. The qNMR method reported by Castilho et al. (2008) was adapted, and the authors pointed out that a standard curve was not necessary, since in NMR the molar equivalence of proton signals is enough for quantification, as reported also by Pauli et al. (2005).

Liu et al. (2010) carried out the quantification with eight *A. annua* extract samples, and the observed values were compared with those obtained with other commonly used techniques: Liquid Chromatography with an evaporative light scattering selector detector (LC-ELSD), Liquid Chromatography coupled to a mass spectrometer (LC-MS), and Thin-Layer Chromatography (TLC). LC-ELSD values showed a fine correlation with qNMR values (with a ± 0.7 mg estimation range, expressed as the differences between both values). The LC-MS had a larger estimation range with respect to qNMR results (between -1.0 and +0.6 mg) and the TLC showed the largest estimation range (between -0.5 to +3.2 mg). It should be noticed that in the two LC methods (LC-ELSD and LC-MS) the separation of artemisinin from other extract components required different conditions (from the columns to the elution protocols), and moreover, the detection devices also varied (ELSD versus MS), hence the small differences in ART values. In the case of the TLC method, the separation was probably worse, and hence the content values were not so accurate. The qNMR avoids the need to separate the products from the extract and therefore avoids this source of result variability.

This fact was also pointed out by Kontogianni et al. (2020), who used 2-D NMR techniques such as ^1^H–^13^C heteronuclear single quantum coherence (HSQC), and ^1^H–^13^C heteronuclear multiple bond correlation nuclear magnetic resonance (HMBC), as well as 1-D total correlation spectroscopy (TOCSY) for the quantification of artemisinin and several other components in a crude diethyl ether extract. The NMR results closely matched those obtained with HPLC with diode-array detection (e.g., 180.75 ± 3.61 mg ART/g dry extract versus 180.32 ± 2.17 mg ART/g dry extract, respectively). The comparison could only be made for those compounds whose standards were available for HPLC.

This qNMR method is accurate and operationally simpler than LC methods and is useful to quantify several components in the same extract, but nevertheless requires a solid training in 1-D and 2D-NMR methods. The qNMR method with internal reference requires a very simple training, and if an “universal” reference was found, it could be used for massive analysis of samples from different origins.

**Figure 1:**
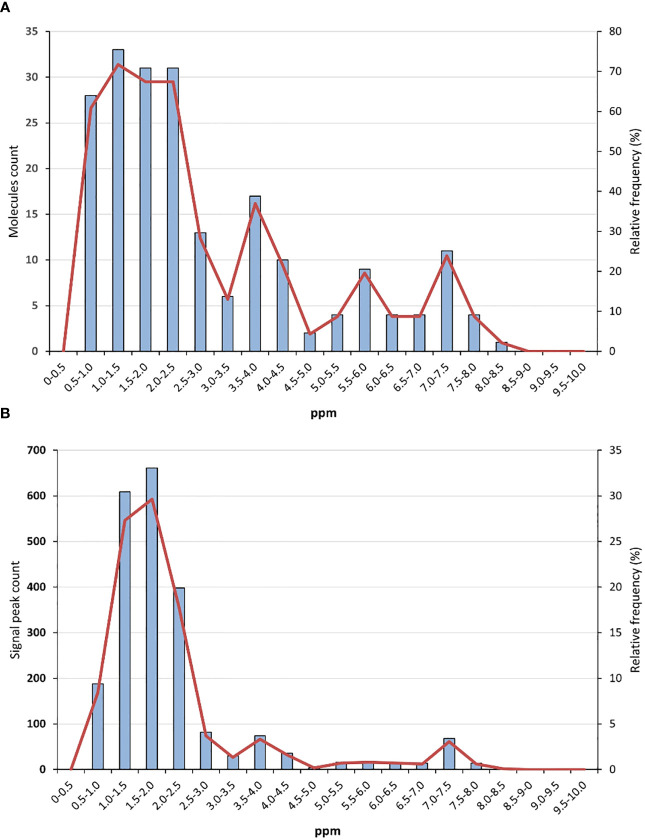
**(A)** Histogram with the number of molecules and their relative frequency in each NMR region, and **(B)** histogram with the number of recorded signals and their relative frequency in each NMR region. The histograms include compounds whose ^1^H (^1^H-^1^H) NMR spectra were run in CDCl_3_ and referenced to the solvent or TMS. The NMR regions are separated by 0.5 ppm. Data were obtained from the natural product database NP-MRD (https://np-mrd.org/), accessed August 25, 2023.

To develop such “universal” reference, avoiding overlapping with extract and product signals was essential. The analysis of the natural product database NP-MRD (https://np-mrd.org/) suggests which NMR regions are the less signal-populated (Wishart et al., 2022). A library of compounds whose ^1^H (^1^H-^1^H) NMR spectra were run in CDCl_3_ and referenced to the deuterated solvent or TMS was analysed (for other d-solvents, please see the Supporting Information). The non-experimental (simulated/predicted) spectra, and those of “not usable” quality were discarded. In the frequency histogram represented in **Figure 1A**, the number of molecules having signals in each NMR region (separated by 0.5 ppm) is represented. In the histogram shown in **Figure 1B**, the number of recorded signals for each NMR region is depicted. As can be seen in **Figure 1A**, no natural product molecules present signals in the δ_H_ 0-0.5 range. Between δ_H_ 5.0-7.00, a relatively low number of molecules display signal peaks. On the other hand, **Figure 1B** shows that the δ_H_ 0-0.5 range presents no signals, while the interval 0.5-3.0 is quite populated, and then the relative number of signals is reduced, particularly in the δ_H_ 5.0-7.00 range. Comparing with the data recorded for other solvents, the δ_H_ 0.0-0.5, 6.0-7.00 and δ_H_ > 8.5 ranges seem to be the less populated ones. Therefore, standards whose signals appear in those regions would be desirable, particularly if they present signals in more than one interval. Otherwise, they cannot be considered “universal” references, since in practice different standards may be required for different samples.

As commented, different commercial references have been developed, and for instance the *Trace*CERT^®^ standards have intense, and often sharp signals, at different shifts, and have been used to quantify pure substances and mixtures (Sigma-Aldrich, 2023). However, to find an “universal” NMR reference for the quantification of structurally-diverse natural products in extracts is not trivial (see **Table S1** in Supporting Information).

In effect, some references such as ethylene carbonate, duroquinone, dimethylsulfone, dimethylmalonic acid, malic acid and alcohols have signals in the same range as most plant extracts (δ_H_ 0.5–5) (Castilho et al., 2008; Nahar et al., 2020; Sigma-Aldrich, 2023). The signals of other standards (such as benzoic acid and its esters, phthalates, 1,2,4,5-tetrachloro-3-nitro-benzene, etc) overlap with those of natural products containing aromatic rings or double bonds. Some NMR references display signals in two different ranges, such as thymol or 1,3,5-trimethoxy-benzene, and are more versatile NMR standards, but still their signals corresponding to aliphatic H-C overlap with those of plant extracts, and the signals in the aromatic/olefinic area can interfere with the few differentiated signals of bioactive natural products. Therefore, they are not suitable as “universal” references.

In this communication we report the design and synthesis of a versatile, low-cost NMR reference, suitable to efficiently quantify artemisinin and a variety of natural products in extracts. It would present signals in two “little crowded” regions, δ_H_ 5-7 and δ_H_<0.5, and at least one of them would not overlap with those of the sample. Bioactive terpenes, steroids, alkaloids, aromatic compounds, and quinones, among others, were quantified with this “universal” reference to show the feasibility of this approach.

## 2 Material and methods

### 2.1 Plant material

Plants of *A. annua* were obtained from Biotech Tricopharming Research SL and cultivated in their experimental fields located in San Cristobal de La Laguna (Tenerife, Spain) during summer 2022, and leaves were harvested in early October before flowering. The leaf size was between 3-4 cm. Even more important, the leaves were at the same phenological state (maturity). Since in *A. annua* the bottom leaves are the oldest, and the upper leaves are the youngest, only leaves at medium height of equally tall plants were taken. Leaves from different plant species were harvested in San Cristobal de La Laguna in March 2023 to obtain a model of a complex extract mixture. The leaves were dried at 38 °C and powdered.

### 2.2 Chemicals and standards

Artemisinin (98%) was obtained from Sigma-Aldrich (CAS 63968-64-9), as well as pure vanillin (CAS 121-33-5), thymoquinone (CAS 490-91-5), carvone (2244-16-8), verbenone (CAS 1196-01-6), santonin (481-06-1), sitosterol (83-46-5), dehydroabietylamine (CAS-1446-61-3), quinine (CAS 130-95-0), and gramine (CAS 87-52-5). The reagents sodium hydride (CAS 7646-69-7), pyrrole (CAS 109-97-7) and tert-butyldimethylsilyl chloride (CAS 18162-48-6) were also purchased from Sigma-Aldrich. Commercially available solvents were analytical grade and were purchased from Merck, as well as the deuterated solvents.

For UHPLC-MS quantification, LC-MS grade acetonitrile was obtained from Merck (Darmstadt, Germany), and deionized water was obtained from a Milli-Q system A10 (Millipore, Massachusetts, USA). Formic acid (> 95%) was from Honeywell (New Jersey, USA). To prevent carryover contamination in the laboratory, volumetric glassware was cleaned using a glass reagent from Godax Laboratories (Maryland, USA), while other glassware was heated at 550°C for 4 hours.

### 2.3 Optimized extraction methodology

Different methodologies were tested to obtain higher plant extract amounts. More detail of the solvents used is shown in **Table 1**.

**Table 1:** Comparison of extraction methodologies. Results obtained after filtering off the vegetal material and removing chlorophylls with active carbon.

Extraction method	Solvent	Extract residue
Method A: Sonication of 0.5 g of dried powdered plant for 1 hour in 12.5 ml of solvent.	n-hexane	7.0 mg
	Acetone	10.2 mg
	Toluene	12.7 mg
Method B: 0.5 g of dried powdered plant in 12.5 ml of solvent were extracted for 18h under stirring.	n-hexane	22.2 mg
	Acetone	42.6 mg
Method C: 2 g of dried powdered plant in 50 ml of solvent were extracted for 18h under stirring, affording C.1 sample.	Acetone	83.5 mg

Method A: The solvent (12.5 mL) was added to powdered dried plants (0.5 g) and the mixture was sonicated at room temperature for 1 h. The organic layer was filtered, and the filtrate was treated with active carbon to remove chlorophylls, until disappearance of the green colour. The active carbon was then filtered off. Then the filtrate was evaporated under vacuum, providing the dry plant extract residue.

Method B: The solvent (12.5 mL) was added to powdered dried plants (0.5 g) and the mixture was stirred for 18 h at room temperature, using a magnetic stirrer. The organic layer was filtered, and the filtrate was treated with active carbon as in Method A to obtain the dry plant extract residue.

Method C: Acetone (50 mL) was added to the powdered dried plants (2 g) and the mixture was stirred for 18 h at room temperature, using a magnetic stirrer. The organic layer was filtered, and the filtrate was treated with active carbon as in Method A to obtain the dry plant extract residue.

### 2.4 Preparation of the reference compound *N*-(t-butyldimethylsilyl)pyrrole and determination of its quality as NMR reference

Sodium hydride (60% suspension in mineral oil; 1.20 g, 30.0 mmol, 1.50 equiv.) was added to a solution of commercially available pyrrole (1.40 mL, 20.0 mmol) in dry THF (40 ml), at 0°C and under nitrogen. The mixture was stirred at the same temperature for 15 min, and then allowed to warm to 25°C for 2 h. Then it was cooled back to 0°C and tert-butyldimethylsilyl chloride (3.92 g, 26.0 mmol, 1.30 equiv.) was added. The mixture was allowed to warm to 25°C. and stirred overnight. Then it was poured into water and extracted with EtOAc (3 x 10 mL). The combined organic layers were dried over magnesium sulfate, filtered and concentrated under vacuum. The crude product was purified via column chromatography (n-hexane:AcOEt, 95:5), to give *N*-(tert-butyldimethylsilyl)pyrrole (2.77 g, 77%). Its ^1^H NMR values are in accordance with reported data (Dhanak and Reese, 1986; Caramenti et al., 2017): ^1^H NMR (400 MHz, CDCl_3_) δ 6.80 (s, 2H), 6.33 (s, 2H), 0.89 (s, 9H), 0.43 (s, 6H). ^1^H NMR (600 MHz, CDCl_3_) δ 6.80 (s, 2H), 6.32 (s, 2H), 0.89 (s, 9H), 0.43 (s, 6H).

The quality of this compound as NMR reference was determined with an inversion/recovery experiment using a 600 MHz Bruker spectrometer (see Supporting information), to calculate the T_1_ of the standard signals. The main parameters of the experiment were: scan number = 8, Recycle Delay *d1 =* 50 sc, with a gain *RG* = 114, a pulse width *pw* = 6.57 µsc, an acquisition time *at* = 1,36 sc.

The NMR data were processed with the MestreNova application (Windows versions 14.3.1, Mestrelab Research SL), using the Data Analysis>New>Integral Graphics tool, as instructed in the manual (see Mestrelab Research, 2023). Thus, for δ_H_ = 6.8, G = 0.205161 and T_1 =_ 4,87 sc; for δ_H_ = 6.33, G = 0.527823 and T_1 =_ 1.89 sc; for δ_H_ = 0.44, G = 1.87069 and T_1 =_ 0.53 sc. For a good quality, the total time TT should be superior to 5 x T_1_. Since TT = at + d1 = 51.36 sc, all the standard signals meet the requirement (Thus, 5 xT1 is 24,35 sc for δ_H_ = 6.8; then 9,45 sc for δ_H_ = 6.33, and finally 2,65 sc for δ_H_ = 0.44).

### 2.5 Preparation of NMR samples for quantification of artemisinin in extract residue

Method I: A solution of pure artemisinin or plant extract residue in CDCl_3_ was placed in an NMR tube. Different amounts of the reference (about 15-20 mg) in 0.5 mL of the deuterated solvent were prepared, and then aliquots of each solution (e.g., 20 µL) were added to the NMR tubes with a Hamilton syringe. The NMR tube was vigorously shaken before running the NMR experiment.

Method II: To a flask with the pure compound (first validations) or the artemisinin-containing plant extract residue (10-15 mg) was added CDCl_3_ (0.2 ml), and the solution was placed in a NMR tube using a Pasteur Pipette. The flask was washed with a second addition of CDCl_3_ (0.2 ml), which was also introduced in the NMR tube. A certain amount of the reference was weighed in a small vial (2-3 mg, the amount in itself is not important, but it should be weighed accurately), well dissolved in 0.1 mL CDCl_3_, and added to the NMR tube. The empty flask was washed with more solvent (0.1 mL), which was also placed in the tube. The homogeneity of the solution was secured with a brief vigorous agitation (a Vortex saves work).

The NMR spectra was recorded in a Bruker 400 MHz NMR spectrometer. In the automatic service mode, the NMR experiments (32 scans, 30° pulses to reduce acquisition time), presented the following parameters: Recycle delay d_1 =_ 1 sc, with a gain *RG* = 101, an acquisition time *at* = 4,59 sc, a pulse width *pw* = 8 µsc, and a spectral width *SW* = 17,85.

The FID (Free Induction Decay) data were processed with the MestreNova application (version 14.3.1, and 10.0.1-14719 for artemisinin analysis, Mestrelab Research SL), using the NMR>Processing tools, with the manual and/or automatic phase and baseline corrections (for more details, see Mestrelab Research, 2023).

Selected reference and artemisinin signals (δ_H_ = 6.33, 6.80 and 0.44 for the reference and δ_H_ = 5.86 for artemisinin) were integrated and their areas were compared.

### 2.6 Artemisinin quantification by UHPLC-MS

Artemisinin quantification was made adding 2 ml of acetone (LC-MS grade): ultrapure water (Milli-Q^®^) in a 75:25 v:v ratio to 10 mg of plant extract residue. Then, vigorous Vortex agitation was applied for 1 minute to achieve a homogeneous solution. After preparation of the samples, they were diluted as follows (artemisinin stock solutions).

Artemisinin stock solutions of 1000 mg/L were prepared in acetonitrile of LC-MS grade and used for the preparation of daily working mixtures of analytes by dilution. All solutions were stored in the dark at -18°C.

With respect to the apparatus and software, analyses were carried out in a Waters Acquity UPLC^®^ H-Class, consisting of a sample manager with a flow-through needle and a quaternary solvent from Waters Chromatography. The UHPLC system was hyphenated with an MS Xevo TQD (Waters Chromatography) with an electrospray ionization interface in positive mode. Mass-lynx™ software from Waters Chromatography was used to control the pumps and sample manager, as well as MS parameters and the collection and processing of spectrum data. Separation was performed on an ACQUITY UPLC HSS T3 column (100 mm x 2.1 mm, 1.8 µm) using a pre-column with the same stationary phase (5 mm x 2.1 mm, 1.8 µm), both from Waters Chromatography. The column and pre-column temperature were set at 40°C.

The mobile phase consisted of acetonitrile (ACN; solvent A) and water (solvent B), both containing 0.1% (v/v) of formic acid. The composition was initially set at 50/50 (v/v) A/B and maintained for 4.5 min. Then, it was changed to 100% A in one min, which was held during one min. Finally, the initial conditions were set up in one min and maintained for another min until the system stabilized. The flow rate was 0.5 mL/min, and the injection volume was 5 µL at 10°C.

The MS analysis was performed in multiple reaction monitoring mode using the retention time and two different transitions as identification points. A maximum tolerance of ±20% for the relative intensity of confirmation to quantification ions with respect to the reference ion ratio was established (European Commission, 2002). The source conditions were as follows: capillary voltage 3.2 kV, source temperature 150°C, desolvation temperature 600°C, cone gas (N_2_) flow rate 120 L/h, desolvation gas (N_2_) flow 1000 L/h, collision gas (Ar) pressure 0.5 bar. The multiple reaction monitoring transitions (MRM), cone voltage, and collision energies applied for artemisinin are shown in **Table S1** (Supporting Information).

A statistical analysis was performed to compare qNMR and UHPLC-MS results in R Studio (version 2022.07.1 + 554,^©^ 2009-2022 RStudio, PBC). Thus, a Wilcoxon signed-rank test was applied in 5 quantified samples.

## 3 Results and discussion

### 3.1 Development of the NMR reference

**Figure 2:**
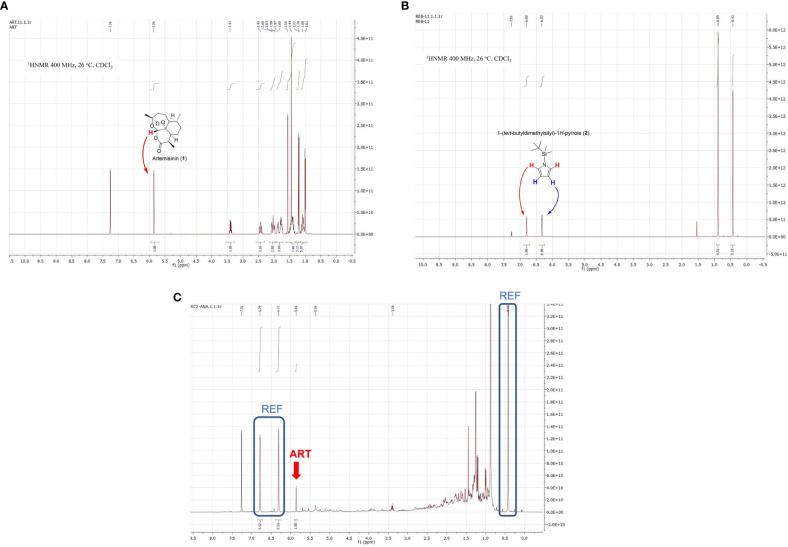
**(A)** ^1^H NMR spectrum of pure artemisinin (1); **(B)** ^1^H NMR spectrum of reference compound TBS-pyrrole (2), and **(C)** ^1^H NMR spectrum of an *Artemisia annua* extract mixed with the reference (REF) compound (key artemisinin signal shown by red arrow as ART).

An ideal NMR reference should present high purity, be inert towards the compounds in the mixture, and have only a few signals that do not overlap with those of the extract and the natural product (Selegato et al., 2019). As illustrated for artemisinin and artemisinin-containing extracts (**Figures 2A, C**, see ampliations in the Supporting Information), the reference substance should have signals only above δ_H_ = 6 and/or below δ_H_ = 1.0. Moreover, since the reference should be “universal”, no signals in the aromatic area (δ_H_ > 7) were desired. These requirements leave a narrow shift range (δ_H_ = 6-7 and δ_H<_1.0) for the internal standard. Relatively few natural products have signals at δ_H_ = 6-7, and only semisynthetic derivatives present them at δ_H_<0.6, but even so, to avoid any risk of overlapping in one of these intervals, reference signals should appear in two or more ranges.

We designed several compounds and decided on N-TBS-pyrrole (2, **Figure 2B**; Dhanak and Reese, 1986; Caramenti et al., 2017). Although this compound was known, this would be its first use as an NMR standard. N-TBS-pyrrole (2) has two multiple bond signals (resembling singlets) above δ_H_ = 6.0, and two alkyl signals in the opposite side of the spectrum. One of them corresponds to two methyl groups attached to silicon, and therefore appears at an unusual high field (δ_H_ = 0.44). Since the reference signals at δ_H_ = 6-7 and δ_H_ = 0.44 are so far apart, and appear in regions with few signals, complete reference/sample overlap is extremely unlikely. In addition, the baseline and phase of the spectrum (which should be optimized to achieve accurate signal areas) can be easily corrected by comparing the relative areas of one signal at δ_H_ = 6-7 (2H) and the signal at δ_H_ = 0.44 (6H), which should be in a 1:3 ratio.

Moreover, in the unlikely case that key signals of other target natural products appeared in the δ_H_ = 6-7 interval, and besides, overlapped the sharp standard references, the quantification would still be possible. Thus, the signal at δ_H_ = 0.4 (6H) could be used to determine the area of the reference singlets at δ_H_ > 6.0 (2H each). Then, the area of the product signal/s could be determined by subtraction.

**Scheme 1:**
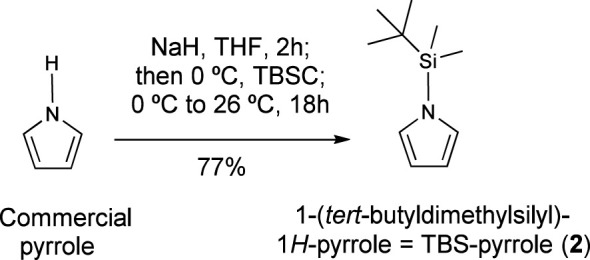
One-step synthesis of “universal” reference TBS-pyrrole from commercial pyrrole.

As additional advantages, N-TBS-pyrrole (2) is readily prepared in one step from inexpensive commercial reagents (**Scheme 1**), so its final cost is quite competitive with respect to commercial references. Finally, it is a liquid with a moderate boiling point that ensures accurate handling under standard conditions, but also removal under high vacuum for 1 hour at least, allowing the recovery of valuable natural products from the quantification sample.

### 3.2 Quantification of artemisinin in samples. Comparison between UHPLC and NMR results

As commented before, both the culture conditions of *Artemisia annua* and the extraction methodology influence artemisinin yields (Ferreira et al., 2005; Jha et al., 2011; Nahar et al., 2020). Therefore, different extraction protocols were compared to find the more suitable for product quantification and for its commercial isolation (Christen and Veuthey, 2001; Lapkin et al., 2006; Castilho et al., 2008; Nahar et al., 2020).

Under some reported protocols the plants were extracted with toluene; however, this aromatic solvent has a high boiling point, and even carrying out the elimination of the solvent under vacuum, a relatively high temperature is needed to remove traces, probably resulting in losses of thermolabile artemisinin (Li et al., 1981; Lin et al., 1986). Other extraction solvents have been reported, such as hexane, diethyl ether, chloroform and acetone (Christen and Veuthey, 2001; Lapkin et al., 2006; Castilho et al., 2008; Nahar et al., 2020). Halogenated solvents are generally avoided since traces of them could be carcinogenic on the long term. Several of these methodologies are compared in **Table 1**, using different solvents and extraction methodologies.

N-hexane and acetone provided the cleanest NMR spectra. The best extract amounts were obtained with low-cost and non-toxic acetone, which is an excellent solvent both for polar and non-polar compounds. In addition, acetone has the lowest boiling point, which allows extraction of thermolabile compounds such as artemisinin in good yields (Castilho et al., 2008; Kontogianni et al., 2020).

Once the preferred methodology was established (last entry in **Table 1**, sample C1), similar amounts (2 g) of four dried powdered *A. annua* plants were extracted (**Table 2**). An average amount of 110 mg of extract was obtained, but as expected, the amount of extract varied with the sample, with C4 providing 40% more extract than C3.

**Table 2:** Extract weight variation in different plants obtained using the same method as for C1 (Method C).

Sample	Weight of dried powdered *A. annua*	Extract residue
C.1	2.0051 g	83.5 mg
C.2	2.0049 g	109.4 mg
C.3	2.0529 g	87.9 mg
C.4	2.0165 g	125.9 mg
C.5	2.0252 g	117.3 mg

In addition, the amount of artemisinin for the same amount of extract also varied. Five extracts from different plants (including sample C1) were prepared according to Method C, and their artemisinin content was determined by UHPLC-MS/MS (**Table 3**). The data of the HPLC results will be later compared with those obtained with the NMR methodology.

**Table 3:** Percentage of artemisinin in different *A. annua* extracts, determined by UHPLC-MS.

Sample	Extract residue (mg)	Artemisinin content (%) ± uncertainty
E.C.1	8.5	17.54 ± 1.07
E.C.2	11.2	14.66 ± 0.79
E.C.3	11.3	15.57 ± 0.79
E.C.4	13.6	11.76 ± 0.65
E.C.5	10	19.01 ± 0.91

Once the extraction methodology had been established, a rapid, simple and low-cost quantification of artemisinin in extracts was addressed, using Nuclear Magnetic Resonance. The accuracy of the methodology was first studied by mixing known amounts of pure artemisinin (1; ART) and the reference (2; REF). The relationship between the amounts (in mmols) of artemisinin and the reference can be extrapolated by determining the NMR areas of one proton of artemisinin and one proton of the reference. Since each signal of the reference at δ_H_ > 6.0 corresponds to two protons, while the signal of artemisinin corresponds to one proton, the area of the reference is halved (alternatively, the artemisinin area is doubled). These relationships are expressed in the Equation 1 below:


$$\frac{mmol\;ART}{mmol\;REF}=\;\frac{area\;1H\;ART}{area\;1H\;REF}=\;\frac{area\;ART\;\times 2}{area\;REF}$$

Equation 1 can be adapted to calculate the amount of artemisinin in milligrams since the MW of artemisinin and the reference are 282.33 and 181.29 respectively. Thus, [Eq1] can be transformed into [Eq2]:


$$\frac{m\text{g}\;ART}{282.33}=\;\frac{m\text{g}\;REF}{181.29}\;\times \;\frac{area\;ART\;\times 2}{area\;REF}$$

**Figure 3:**
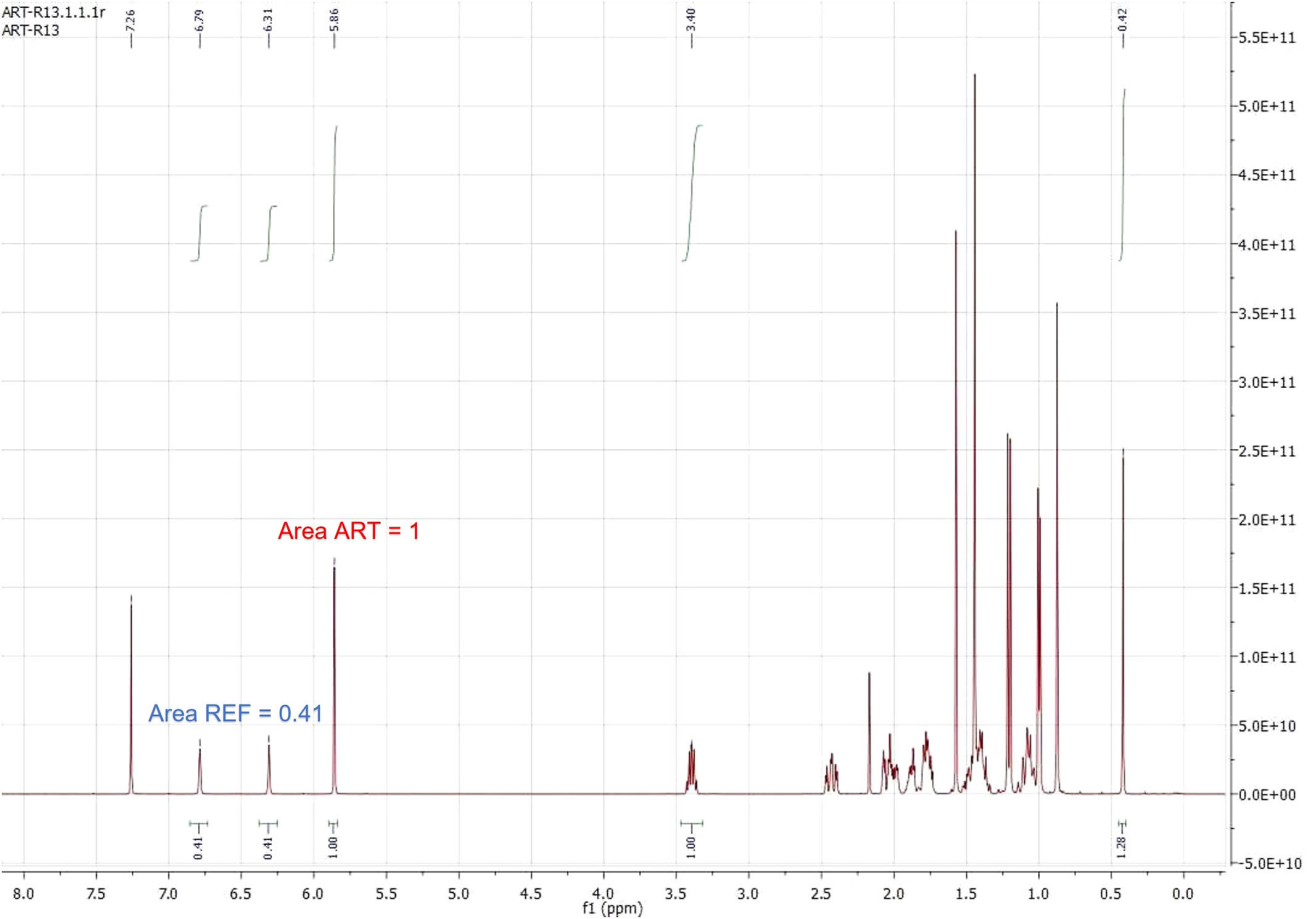
^1^H NMR spectrum of a mixture of pure artemisinin (ART) and the reference (REF) with area values of key signals. ^1^HNMR in CDCl_3_, 400 MHz, 26 °C.


The example shown in **Figure 3** shows that even if the ratio of reference and artemisinin is quite different, the NMR method gives reliable results. Thus, the artemisinin key signal (right one) was given a 1.0 a.u. value, and the reference signals thus obtained a 0.41 a.u. value. The application of Equation 2, for a known amount of reference (0.6 mg), gave the amount of artemisinin (4.56 mg), which matched well with the real one (4.55 mg):

$$\frac{m\text{g}\;ART}{282.33}=\;\frac{0.6\;m\text{g}\;REF}{181.29}\;\times \;\frac{2.0}{0.41}$$

Other examples with different amounts of artemisinin and reference are presented in **Table 4** (more detailed in Supporting Information), with very good results in most cases, even with small amounts of artemisinin (2.2, 1.6 and 0.8 mg).

**Table 4:** A known amount of pure artemisinin calculated by NMR with Methods I and II.

Method	REF (mg)	$\frac{Area\;ART\;\times \;2}{Area\;REF}$	Artemisinin by NMR quantification (mg) ± relative error	Real amount of artemisinin (mg)
I	0.60	4.88	4.56 ± 0.00	4.55
I	1.20	2.44	4.56 ± 0.00	4.55
I	1.70	1.69	4.49 ± 0.01	4.55
II	3.70	0.43	2.46 ± 0.11	2.20
II	2.20	0.50	1.72 ± 0.07	1.60
II	2.50	0.20	0.78 ± 0.03	0.80

In these first validations shown in **Table 4**, two procedures were compared. In the first (Method I), a solution of the reference in 0.5 mL of the deuterated solvent was prepared, and then different aliquots of this solution were used for the experiments. This method (I) has the advantage that initially a relatively large amount of reference could be weighed (15-20 mg), minimizing weighing errors, and then a small aliquot can be extracted, allowing to accurately measure a small amount of reference (such as the 0.6 mg shown in the example of **Figure 3**). However, the method required careful measuring of the final solution volume, which was the sum of the liquid reference substance and the deuterated solvent. In addition, to measure the volume of the aliquot with precision required Hamilton syringes. Despite this, this method is preferable if small amounts of the reference are needed.

The second methodology (Method II) is operationally simpler. As commented, the artemisinin (or the extract) is weighed in a vial, dissolved in the deuterated solvent, and introduced in the NMR tube; the vial is washed once to ensure completed addition. The reference is weighed in another vial and the procedure is repeated. The amount of reference and artemisinin/extract can vary as desired; the only important precaution is to weigh accurately the reference with a precision balance (which under good calibration can have a precision of ± 0.2 mg). The amount of solvent does not need to be precise since the final reference/compound ratio would be similar. Therefore, the method is operationally simple and allows processing many samples in a small time.

Once that the preliminary methods were validated with pure samples, we studied the influence of the extract in the NMR determinations. The signals of the extract could overlap with the key artemisinin and (to a lesser extent) the reference signals, hindering the precise calculation of signal areas. To have as many extract signals as possible, a model vegetal extract (not containing artemisinin) was prepared using plants from different species. The stability of added standard artemisinin in the model extract (and later natural one in the *A. annua* extract) including the reference was also checked. The relationship between artemisinin and the reference signals was not altered, even after 3 days in the NMR tube.

**Figure 4:**
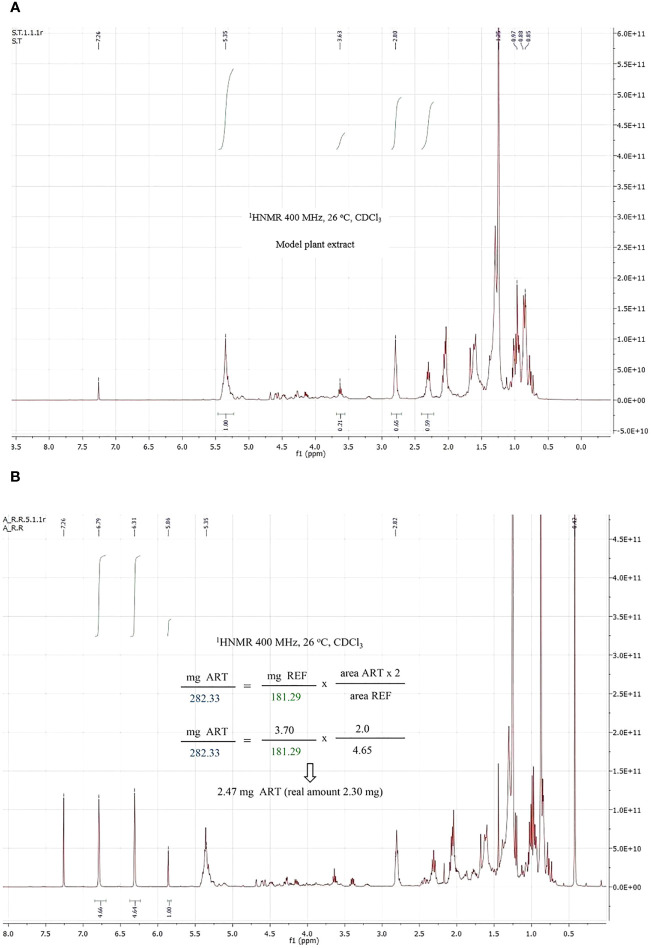
^1^H NMR spectrum of a plant model extract **(A)** and a mixture of pure artemisinin, the reference compound and the plant model extract **(B)**.

Then an accurate amount of artemisinin and the reference were mixed with the model extract, and the amount of artemisinin was again calculated using method II, as detailed in the Supporting Information. **Figure 4** shows the plant extract (**4A**) and mixture of the extract, pure artemisinin and reference (**4B**). To our satisfaction, the reference signals did not overlap with those of the extract. Using a random amount of the reference (3.70 mg) and measuring the areas (1.0 for the artemisinin key signals and 4.65 for the reference) before applying the above formulas, an artemisinin amount of 2.47 mg was calculated, which was close to the real one (2.30 mg).

**Figure 5:**
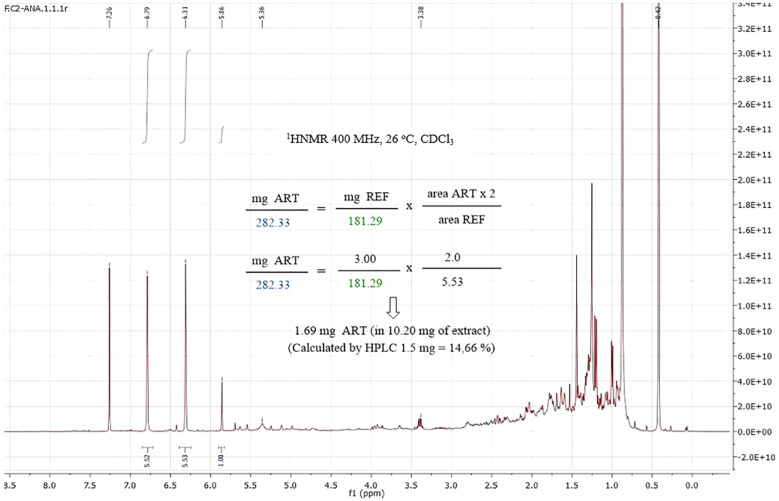
^1^H NMR spectrum of an *Artemisia annua* extract with the reference, and quantification of artemisinin.

Finally, the quantification of artemisinin was carried out in real *A. annua* extracts to compare the results with those obtained with UHPLC-MS. As shown in **Figure 5**, in the ^1^HNMR spectra of the *A. annua* extract, the artemisinin signal at δ_H_ = 5.86 is clearly separated from the others. Moreover, this signal is an easy-to-integrate singlet. Other artemisinin signals can overlap with those in the extract, and therefore, are not so appropriate for quantification. A good correlation between the NMR and UHPLC results was obtained herein and for the other samples EC1-5 described in the Supporting Information, as summarized in **Table 5**. The differences are less than 0.5 mg, generally in the range 0.1-0.4 mg. In general, the qNMR values were slightly higher than HPLC values, similarly to the results reported for LC-ELSD by Liu et al. (2010), which could be due to several factors. Unlike HPLC or LC, qNMR does not require separation of the extract components along a column, which minimizes small loses by retention in the column support. The type of HPLC detector can also generate small differences as shown by Liu et al. (2010).

**Table 5:** Artemisinin calculated by NMR and UHPLC-MS in *A. annua* plant extract.

Sample	REF (mg)	$\frac{Area\;ART\;\times \;2}{Area\;REF}$	Extract residue for NMR quantification (mg)	Artemisinin by NMR quantification (mg) ± relative error	Artemisinin by UHPLC-MS quantification (mg)
E.C.1	0.66	4.25	23.70	4.37 ± 0.05	4.16
E.C.2	3.00	0.36	10.20	1.69 ± 0.11	1.50
E.C.3	2.90	0.39	9.00	1.74 ± 0.17	1.44
E.C.4	2.80	0.56	16.30	2.45 ± 0.22	1.92
E.C.5	2.60	0.47	9.10	1.89 ± 0.08	1.73

As commented before, overlapping of the NMR and HPLC signals with those of minor impurities can also affect the values. This overlapping could result in slight over or underestimation of the real signal areas. While in HPLC there is overlapping of compound signals (due to separation problems between artemisinin and related terpenes, and even non-related compounds of similar polarity), in NMR there is overlapping of proton signals. To solve this problem, in the first case, the column and/or the elution system is optimized, in the second case, a change of d-solvent usually gives good results (as shown later).

Finally, artemisinin mean value in *A. annua* plant extract residues ($\bar{X}$ ± SE) by qNMR was 1.13 ± 0.50 mg of ART which was not significantly different to UHPLC-MS result (1.14 ± 0.51 mg of ART).

### 3.3 Quantification of structurally-diverse natural products in a model vegetal extract, using the “universal” NMR reference

**Figure 6:**
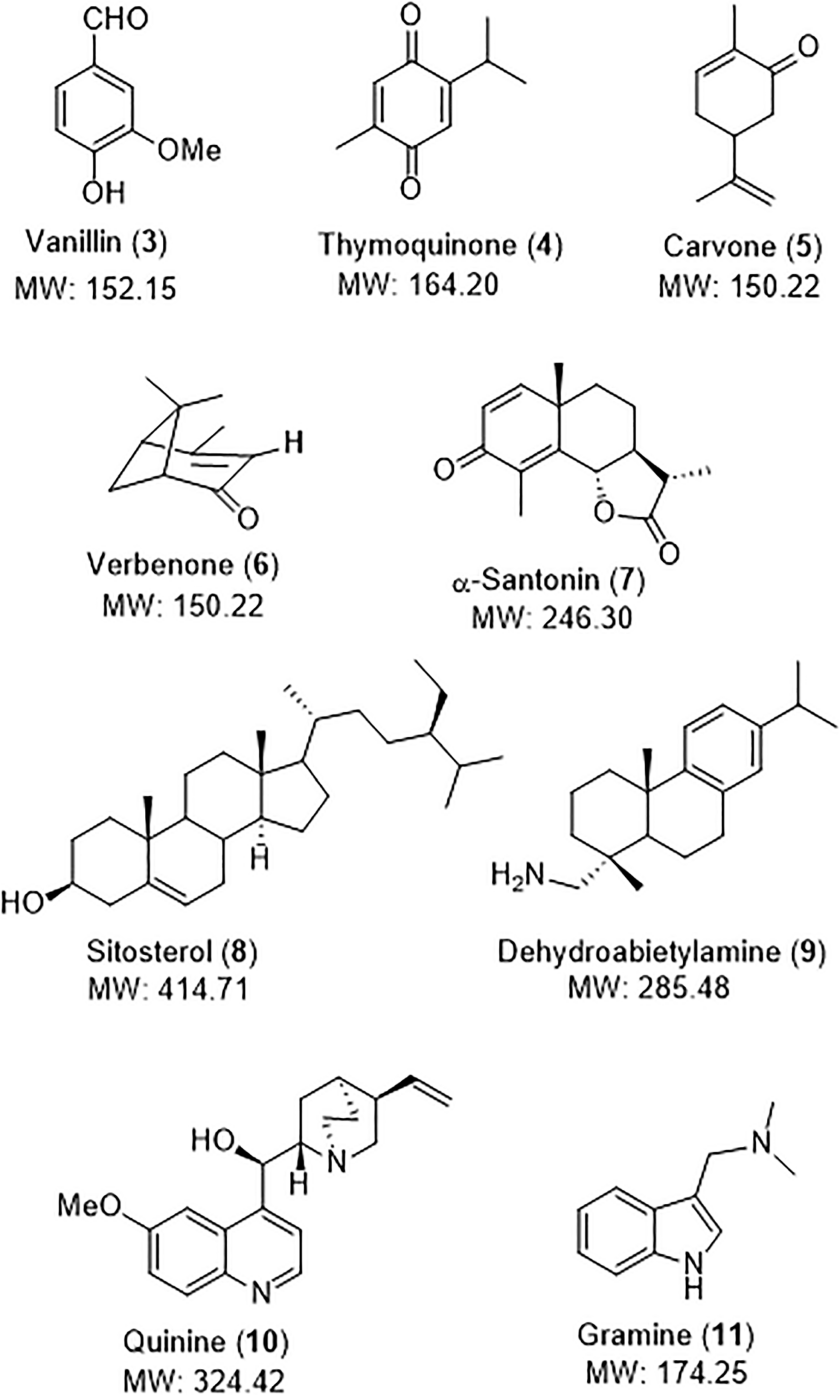
Structurally-diverse natural products for quantification in a model vegetal extract.

To prove the utility of the NMR reference, it was used to quantify a library of structurally-diverse natural products of commercial importance, which are shown in **Figure 6**. Thus, vanillin (3) is an example of aromatic natural product and is found in the extract of the vanilla bean. It is widely used as flavouring in foods and drugs (Dignum et al., 2001; Walton et al., 2003). Thymoquinone (4) was isolated from *Nigella sativa*, and displays cardioprotective, antidiabetic and anti-inflammatory properties (Farkhondeh et al., 2017; Leong et al., 2021). The terpenes carvone (5), verbenone (6) and santonin (7) are also bioactive products. Carvone (5) is found in the essential oils of spearmint and caraway seeds, and displays antibacterial and antifungal activity (De Carvalho and Da Fonseca, 2006). The pleasant-smelling verbenone (6) is found in a variety of plants, and since it also acts as an insect pheromone, it is used for pest control (Progar, 2005; Martini et al., 2020). Santonin (7) is another component of the *Artemisia* genus, and has been used for its antihelmintic (roundworms) properties, and also displays antibacterial and antipyretic activities (Sakipova et al., 2017). Sitosterol (8) is a phytosterol used in food additive E499 (Patel and Thompson, 2006; Food Standards Agency, 2023), while dehydroabietylamine (9, also called leelamine) is a diterpene amine which acts as inhibitor of pyruvate dehydrogenase kinase, and also presents promising antitumoral activity (Aicher et al., 1999; Jung et al., 2022). Leelamine has also served as precursor of several alkaloids. Examples of alkaloids are quinine (10) and gramine (11). Quinine, an alkaloid from *Cinchona* sp., is an antimalarial drug, a food additive and a fine chemical, as commented in the Introduction. Gramine (11) is an indole alkaloid found in several plant species mostly in Gramineae species, such as *Hordeum* (barley) and *Phalaris* grasses, the giant reed *Arundo donax*, or the silver maple *Acer saccharinum* (Pachter et al., 1959; Leete and Minich, 1977; Semenov and Granik, 2004; Grün et al., 2005). It probably plays a defensive role, since it is toxic to many insects (Corcuera, 1984). It can be used to used to control undesired algae invasions, specially *Coelastrella* sp. (Grün et al., 2005; Canton et al., 2019).

To validate the utility of NMR quantification, a known amount of these compounds and the reference was mixed with the model vegetal extract. Using procedure II, the amount of the natural product was calculated and compared with the real amount. Excellent results were obtained, as shown in **Table 6** and the NMR spectra described in the Supporting Information. In general, the reference signals did not overlap with the extract or natural product signals.

**Table 6:** Quantification of structurally-diverse natural products in a model vegetal extract by NMR.

Compound	MW	$\frac{Area\;Compound\;}{Area\;REF}$	Compound by NMR quantification (mg) ± relative error	Real amount of compound (mg)
Vanillin (3)	152.15	1.36	2.63 ± 0.12	2.30
Thymoquinone (4)	164.20	1.28	2.43 ± 0.07	2.60
Carvone (5)	150.22	1.49	2.80 ± 0.00	2.80
Verbenone (6)	150.22	1.54	2.93 ± 0.01	2.90
α-Santonin (7)	246.30	1.23	3.67 ± 0.02	3.60
Dehydroabietylamine (9)	285.48	0.68	3.95 ± 0.01	4.00
Quinine (10)	324.42	1.18	4.02 ± 0.03	3.90
Gramine (11)	174.25	1.01	2.52 ± 0.09	2.30

**Figure 7:**
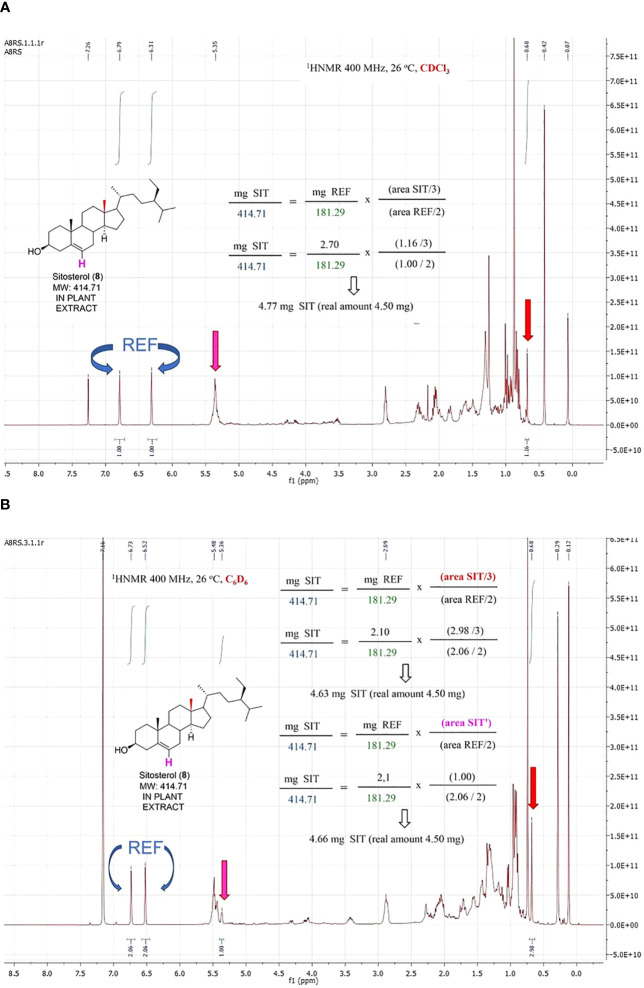
Quantification of sitosterol in a model vegetal extract with CDCl_3_ **(A)** and C_6_D_6_ **(B)** as deuterated solvent.

The quantification of sitosterol (8, **Figure 7**) deserves a detailed comment. When the experiment was run in CDCl_3_, the olefinic signal of sitosterol overlapped with those of the extract. Fortunately, a singlet corresponding to one methyl group at δ = 0.68 separated from the bulk, and quantification was carried out using it. However, the separation of the signals improved by replacing the deuterated solvent. Thus, using d-benzene, the olefinic signal separated from those of the extract, and the resolution of the methyl group also improved, affording better accuracy. Therefore, a change in the d-solvent can be useful to overcome problems due to signal overlapping.

Finally, the quantifications of dehydroabietylamine (9) and the alkaloids quinine (10) and gramine (11) in the model plant extract were carried out. Gramine presented some solubility problems at higher doses, and we considered using other solvent such as d-methanol for this case. However, at the small amounts used in this experiment, the quantification in CDCl_3_ proceeded well, giving accurate results for these nitrogen compounds. Therefore, the feasibility of using *N*-TBS-pyrrole as an universal reference for structurally-diverse natural products in plant extracts was demonstrated.

## 4 Conclusions

A novel “universal” NMR reference was introduced for the quantification of structurally-diverse natural products in plant extracts. This reference, N-TBS-pyrrole (2), is inexpensive and readily prepared in one step from commercial reagents. The molecule is symmetric, with several sets of equivalent protons, and provides intense singlet signals which do not overlap with most of the extract and target compound signals. In addition, it is a liquid with a moderate boiling point, which can be handled precisely during the quantification, but can also be removed from the sample under high vacuum, allowing the recovery of valuable natural products from the quantification sample.

The NMR method was studied first with the antimalarial artemisinin. The results were satisfactory both with known amounts of pure artemisinin and in extracts of *A. annua*, the latter being compared with those obtained by UHPLC-MS which is a standard quantification method for artemisinin. Once the methodology was optimized and validated, it was applied for the quantification of structurally-diverse products such as aromatic compounds, quinones, terpenes, steroids, and alkaloids in a model plant extract. The quantification afforded excellent results.

As commented in the Introduction, a limitation of this technology is that the target compounds should have signals that separate from the bulk, and whose area can be measured accurately. If the separation is not good, then the standard HPLC techniques or NMR/HPLC combinations should be used. However, as demonstrated with artemisinin, sitosterol, and the other library compounds, most bioactive natural products possess some aromatic, olefinic, or heteroaromatic functions, or have differentiated methyl group signals. The signal separation can also be improved in some cases by varying the deuterated solvent. Therefore, this NMR reference and the reported protocol can be applied in most cases.

This low-cost method only needs a small amount of the extract, is accurate and operationally simple, and a large volume of samples can be processed in little time. Indeed, data acquisition is fast (less than 3 min and no calibration curves are needed), particularly with automatic switching of samples placed on NMR carousels.

Therefore, the novel NMR reference and the optimized protocols will be quite useful for natural product chemists and industrial quality controls.

## Data availability statement

The original contributions presented in the study are included in the article/**Supplementary Material**. Further inquiries can be directed to the corresponding author.

## Author contributions

ALGG prepared the extracts supervised by DJA and then performed the NMR quantification experiments supervised by AB; DH synthesized the NMR probe, in collaboration with AB. ÁSM performed the UHPLC experiments. AB and DJA, in collaboration with ALGG, designed and supervised the experiments and wrote the manuscript. All authors contributed to manuscript revision, read, and approved the submitted version.
