# Supplementary material for: TBS-pyrrole as an “universal” reference to quantify artemisinin and structurally-diverse natural products in plants extracts by NMR
Source: Front Plant Sci. 2023 Sep 29;14:1255512. doi: 10.3389/fpls.2023.1255512 (PMC10570554; doi:10.3389/fpls.2023.1255512)
Supplement: Supplementary file 1 [file DataSheet_1.pdf]

Supplementary Material

TBS-pyrrole as an "universal" reference to quantify artemisinin and structurally-diverse natural products in plants extracts by NMR

Ana L. García-García[1,2], Dácil Hernández[1], Álvaro Santana-Mayor[3], David Jiménez-Arias[4,5,*], Alicia Boto[1,*]

*** Correspondence:** Alicia Boto, alicia@ipna.csic.es, David Jiménez-Arias: djimenez@icia.es

Content	Page number
**Figure 1. Frecuency histograms of natural products spectra run in methanol.**	S02
**Table 1. ^{1}H NMR shifts intervals (δ_H) in $CDCl_3$ of commercial and published NMR references.**	S03
**Table 2. QqQ-MS/MS parameters of artemisinin.**	S04
**^{1}H NMR spectra of artemisinin**	S05
**^{1}H NMR spectra of reference compound (REF)**	S06
**First validations: Mixtures of Reference and Artemisinin-Method I**	S07
**First validations: Mixtures of Reference and Artemisinin-Method II**	S10
**Second validations: Mixtures of reference and artemisinin in a model plant extract – Method II**	S13
**Third validations: mixtures of reference and *Artemisia annua* extract-Method I**	S15
**Third validations: mixtures of reference and *Artemisia annua* extract-Method II**	S17
**Validation of Method II to determine other natural products in a model plant extract: vanillin, thymoquinone, carvone, verbenone, α-santonin, sitosterol, dehydroabietylamine, quinine and gramine**	S21

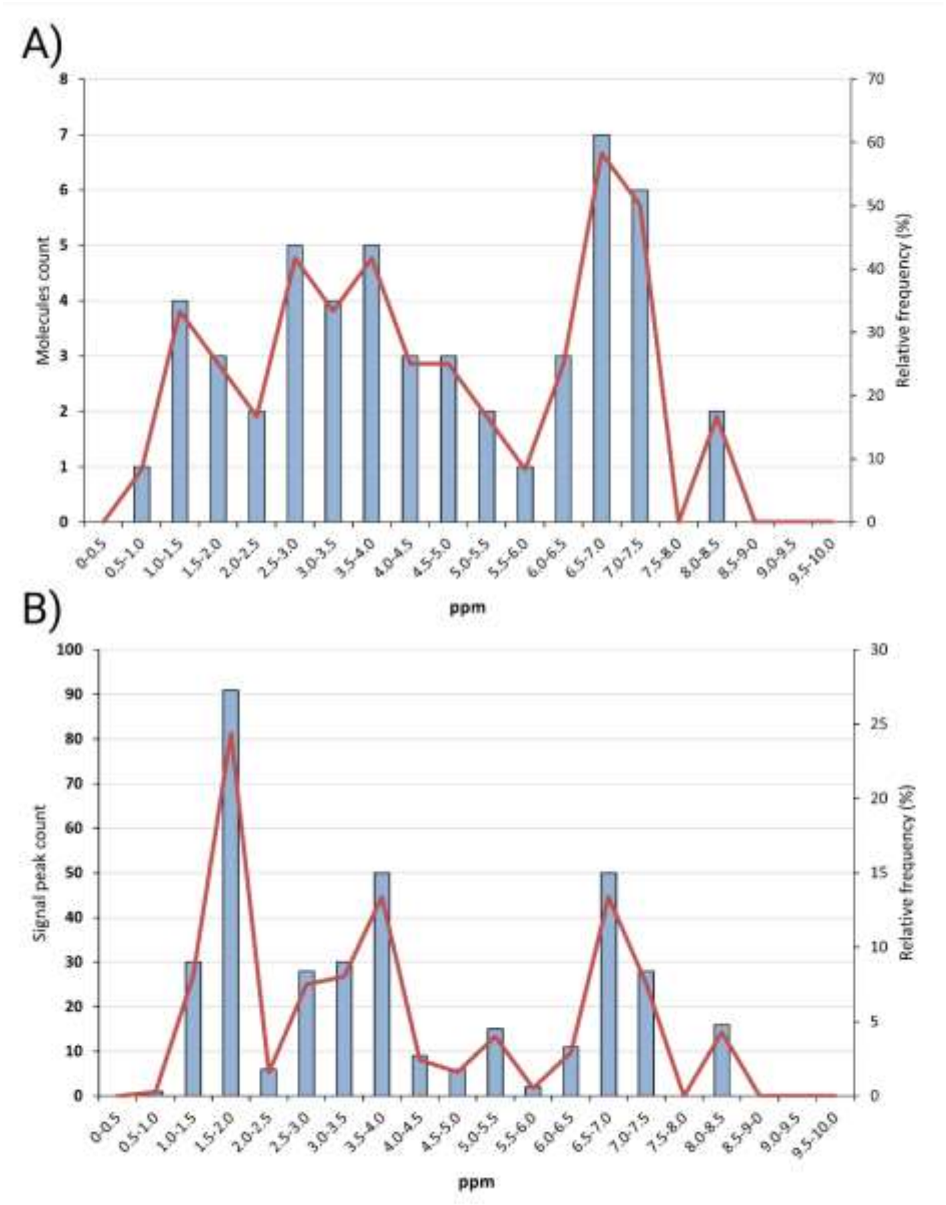

Figure 1. (A) Histogram with the number of molecules and their relative frequency in each NMR region, and (B) histogram with the number of recorded signals and their relative frequency in each NMR region. The histograms include compounds whose ^{1}H (^{1}H-^{1}H) NMR spectra were run in methanol and NMR regions are separated by 0.5 ppm. Data were obtained from the natural product database NP-MRD (https://np-mrd.org/), accessed August 25, 2023

Table 1. ^{1}H NMR shifts intervals (δ_H) in $CDCl_3$ of commercial and published NMR references. The blue background represents crowded signal areas in extracts and bioactive products. [a]Maleic acid is not soluble in d-chloroform, but it has a signal at δ_H 6.3 in d-methanol.

	^{1}H NMR shifts intervals (δ_H) in $CDCl_3$								
**Standard**	**0-0.5**	**0.5-1.0**	**1.0-2.5**	**2.5-4.0**	**4.0-6.0**	**6.0-7.0**	**7.0-7.5**	**7.5-9.0**	**9.0-11.0**
t-butanol	---	---	1.26, 2.01	---	---	---	---	---	---
Maleic acid[a]	---	---	---	---	---	---[a]	---	---	---
Benzoic acid	---	---	---	---	---	---	---	7.5, 7.7, 8.1	---
Benzyl benzoate	---	---	---	---	5.4	---	---	8.1	---
Dimethyl sulfone	---	---	---	3.0	---	---	---	---	---
Dimethyl terephthalate	---	---	---	---	4.0	---	---	8.1	---
Duroquinone	---	---	2.0	---	---	---	---	---	---
Ethylene carbonate	---	---	---	---	4.5	---	---	---	---
Ethyl 4 (dimethyl-amino) benzoate	---	---	1.4	3.1	4.3	6.7	---	7.9	---
Methyl 3,5-dinitrobenzoate	---	---	---	---	4.1	---	---	---	9.2, 9.3
1,2,4,5-Tetramethylbenzene	---	---	2.2	---	---	---	7.0	---	---
1,2,4,5-Tetrachloro-3-nitro-benzene	---	---	---	---	---	---	---	7.8	---
Thymol	---	---	1.3, 2.3	3.2	---	6.6, 6.8	7.1	---	---
1,3,5-Trimethoxybenzene	---	---	---	3.8	---	6.1	---	---	---

Table 2. QqQ-MS/MS parameters of artemisinin.

Retention time (min)	Molar weight (g/mol)	MRM (m/z)	Cone voltage (V)	Collision energy (V)
2.87	282.3	283.17 > 209.08	20	6
		283.17 > 151.09	20	14

^{1}H NMR spectra of artemisinin

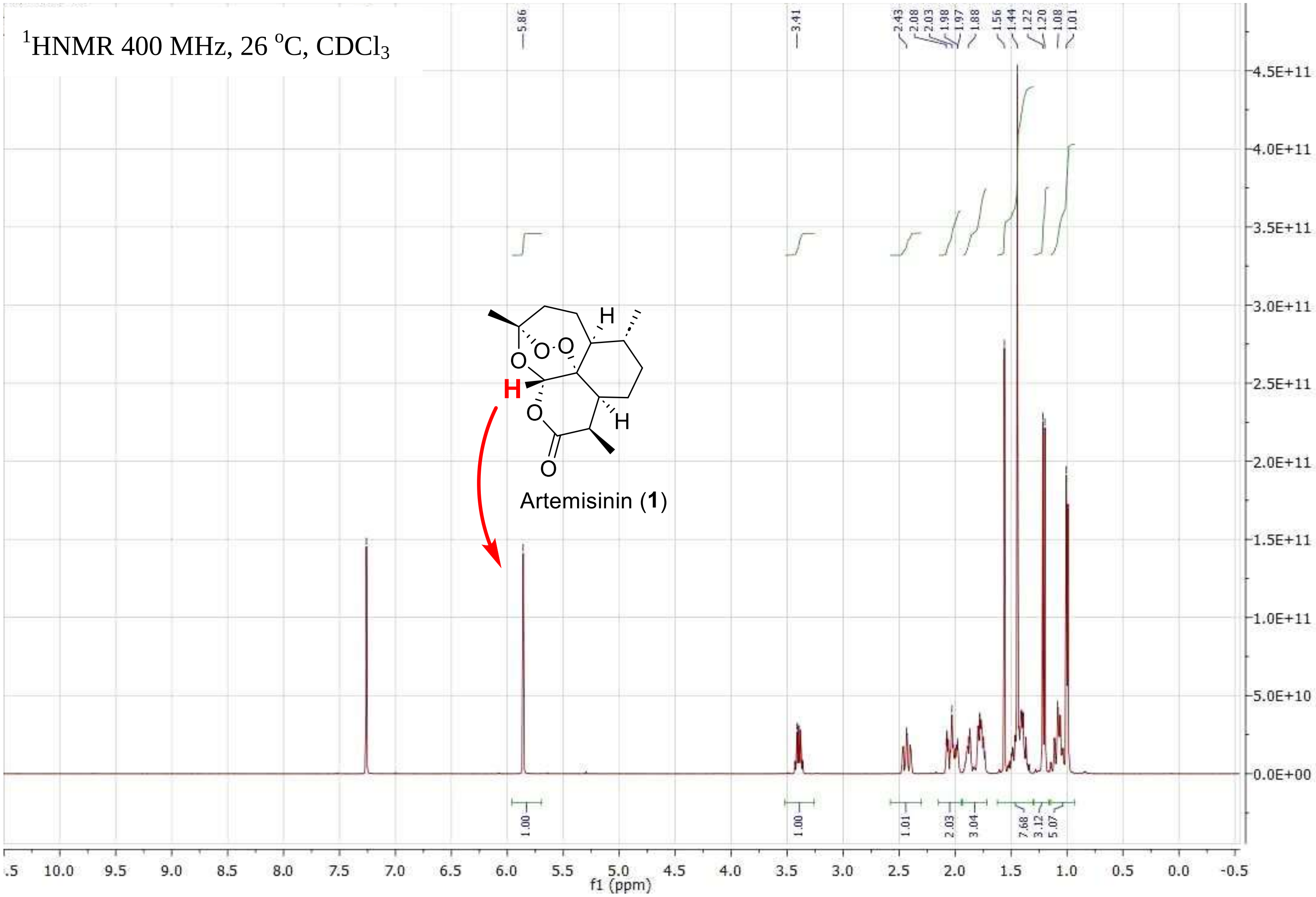

Supplementary Figure 1. ^{1}H NMR spectra of artemisinin.

^{1}H NMR spectra of reference compound (REF)

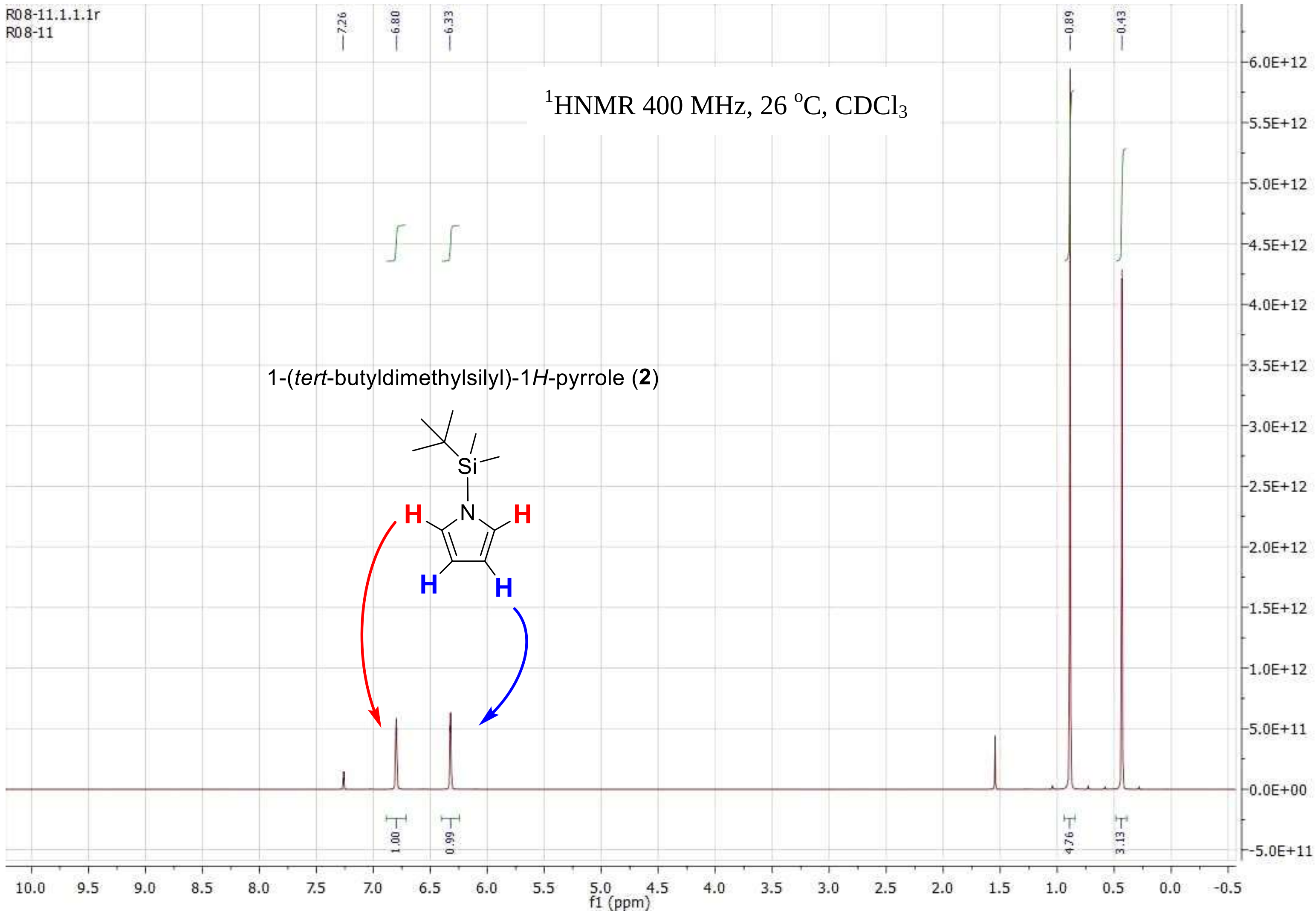

Supplementary Figure 2. ^{1}H NMR spectra of reference compound.

First validations: Mixtures of Reference and Artemisinin-Method I

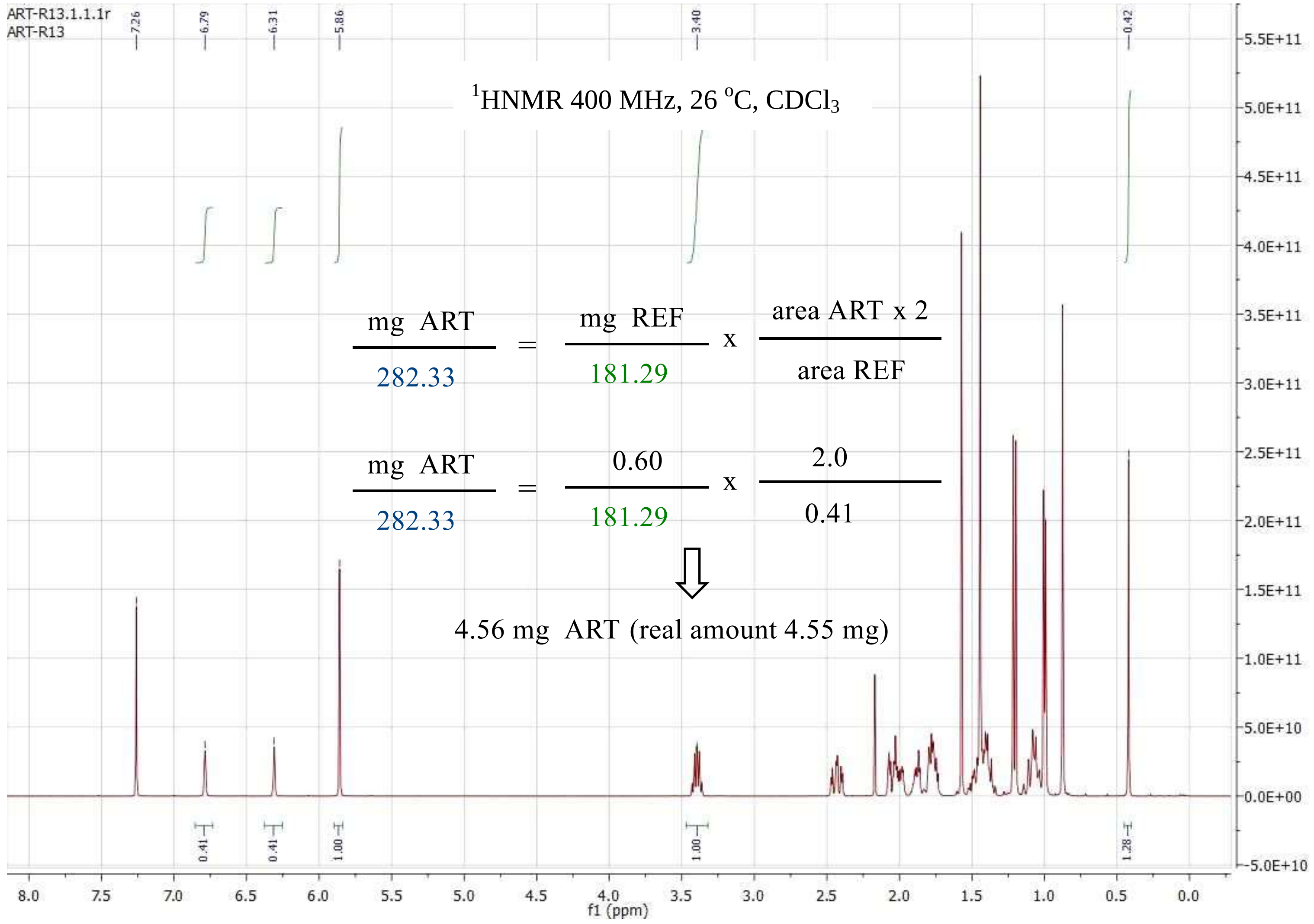

Supplementary Figure 3. First validations: mixtures of reference and artemisinin-method I. ^{1}H NMR spectra 1.

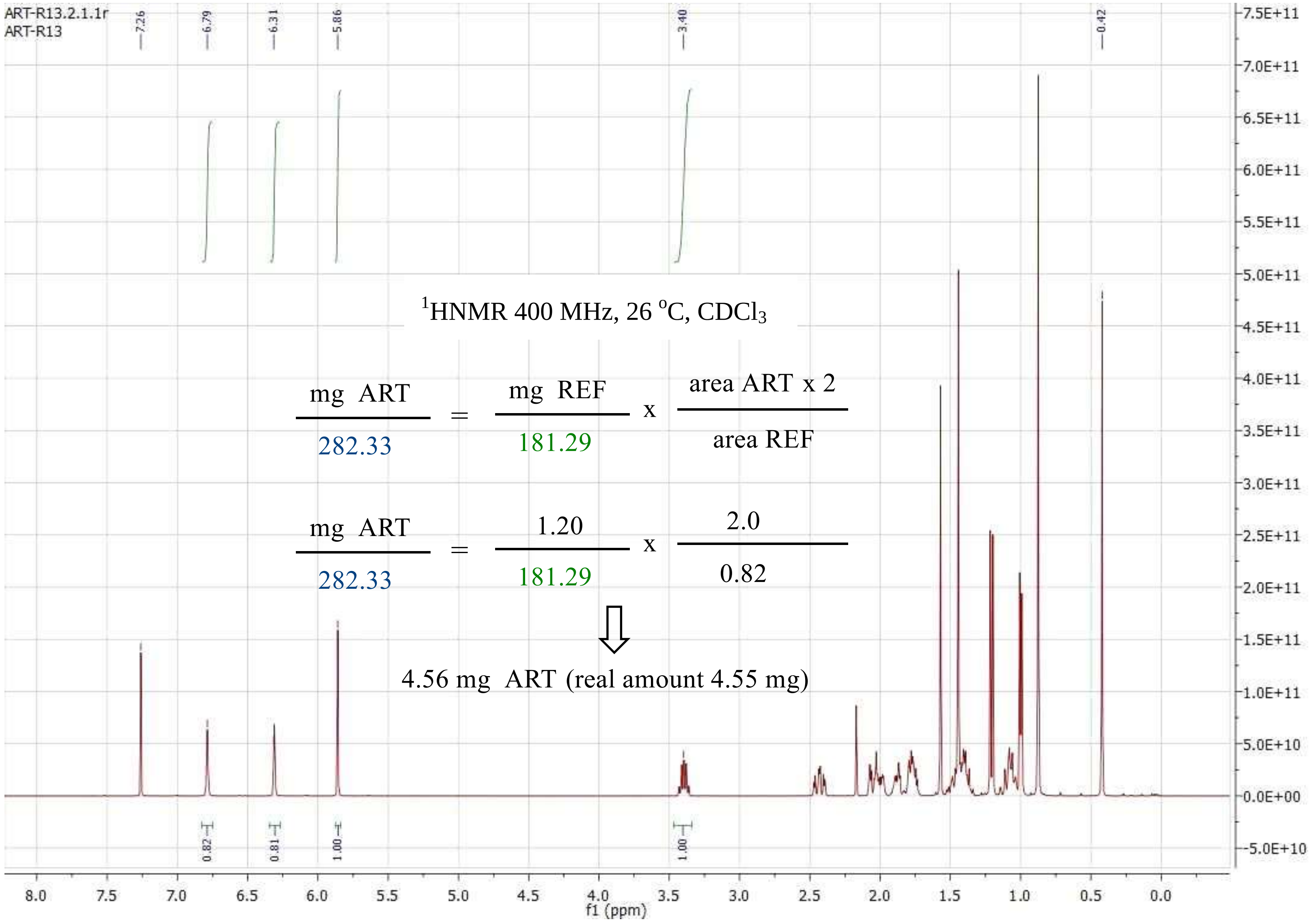

Supplementary Figure 4. First validations: mixtures of reference and artemisinin-method I. ^{1}H NMR spectra 2.

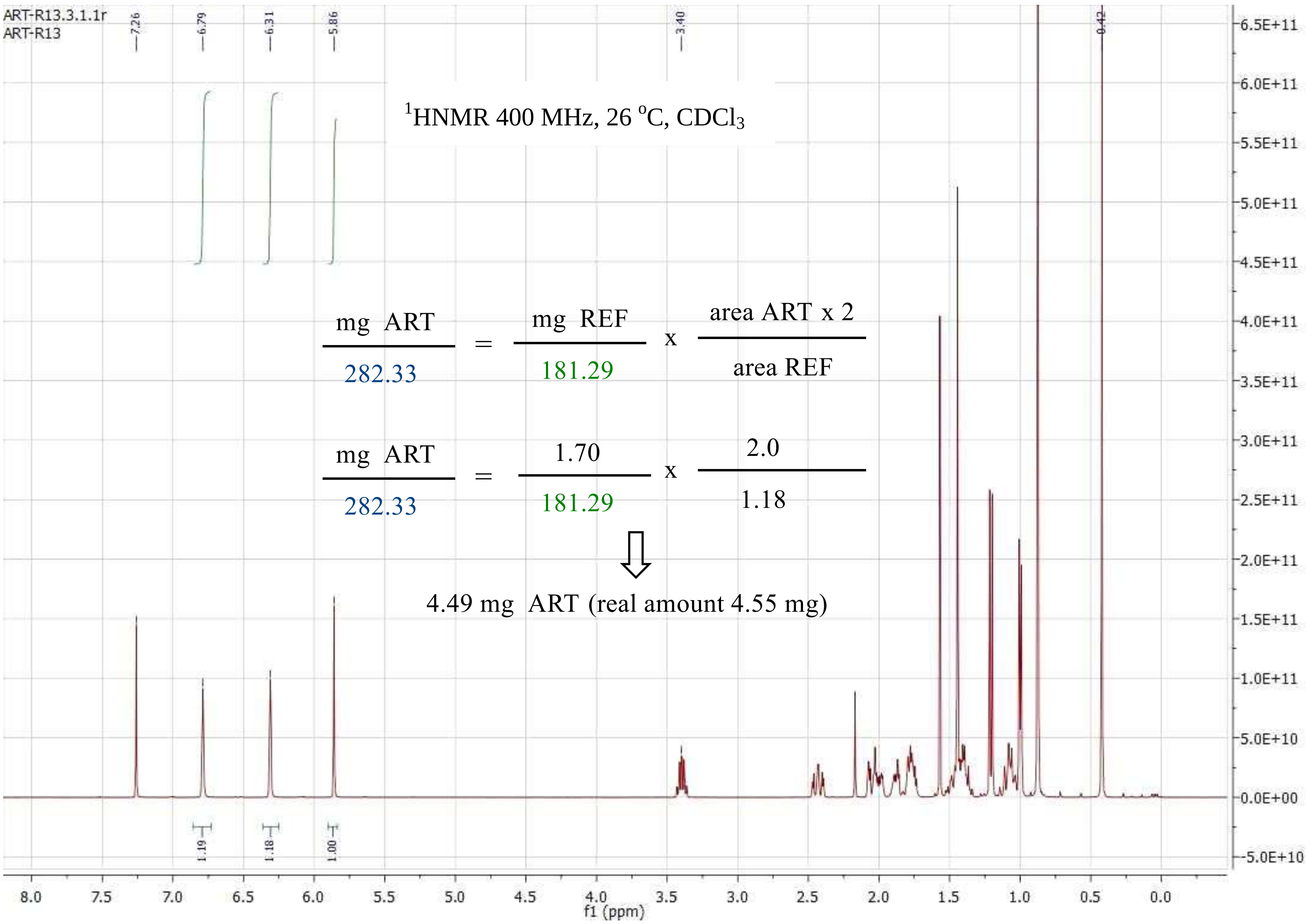

Supplementary Figure 5. First validations: mixtures of reference and artemisinin-method I. ^{1}H NMR spectra 3.

First validations: Mixtures of Reference and Artemisinin-Method II

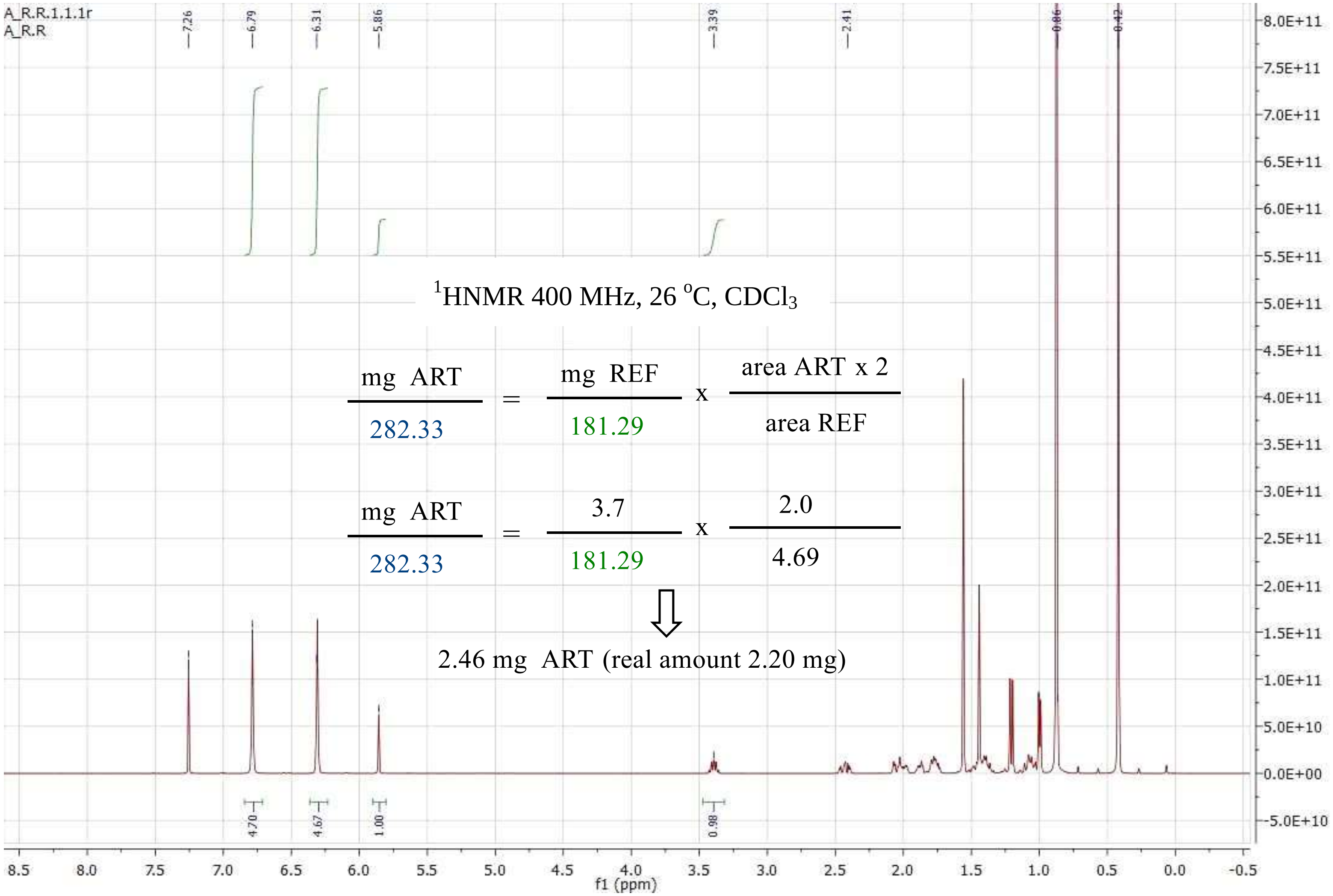

Supplementary Figure 6. First validations: mixtures of reference and artemisinin-method II. ^{1}H NMR spectra 1.

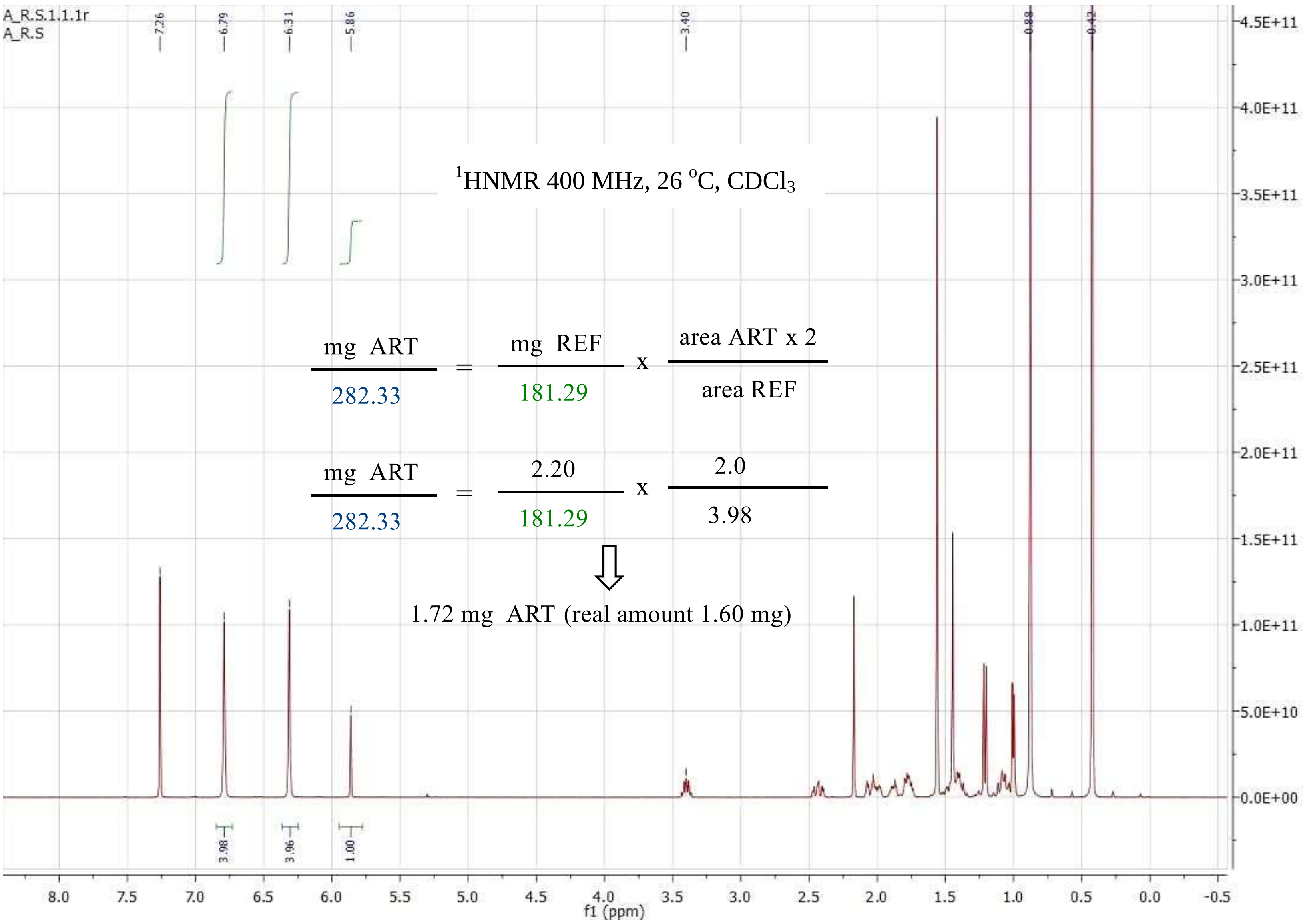

Supplementary Figure 7. First validations: mixtures of reference and artemisinin-method II. ^{1}H NMR spectra 2.

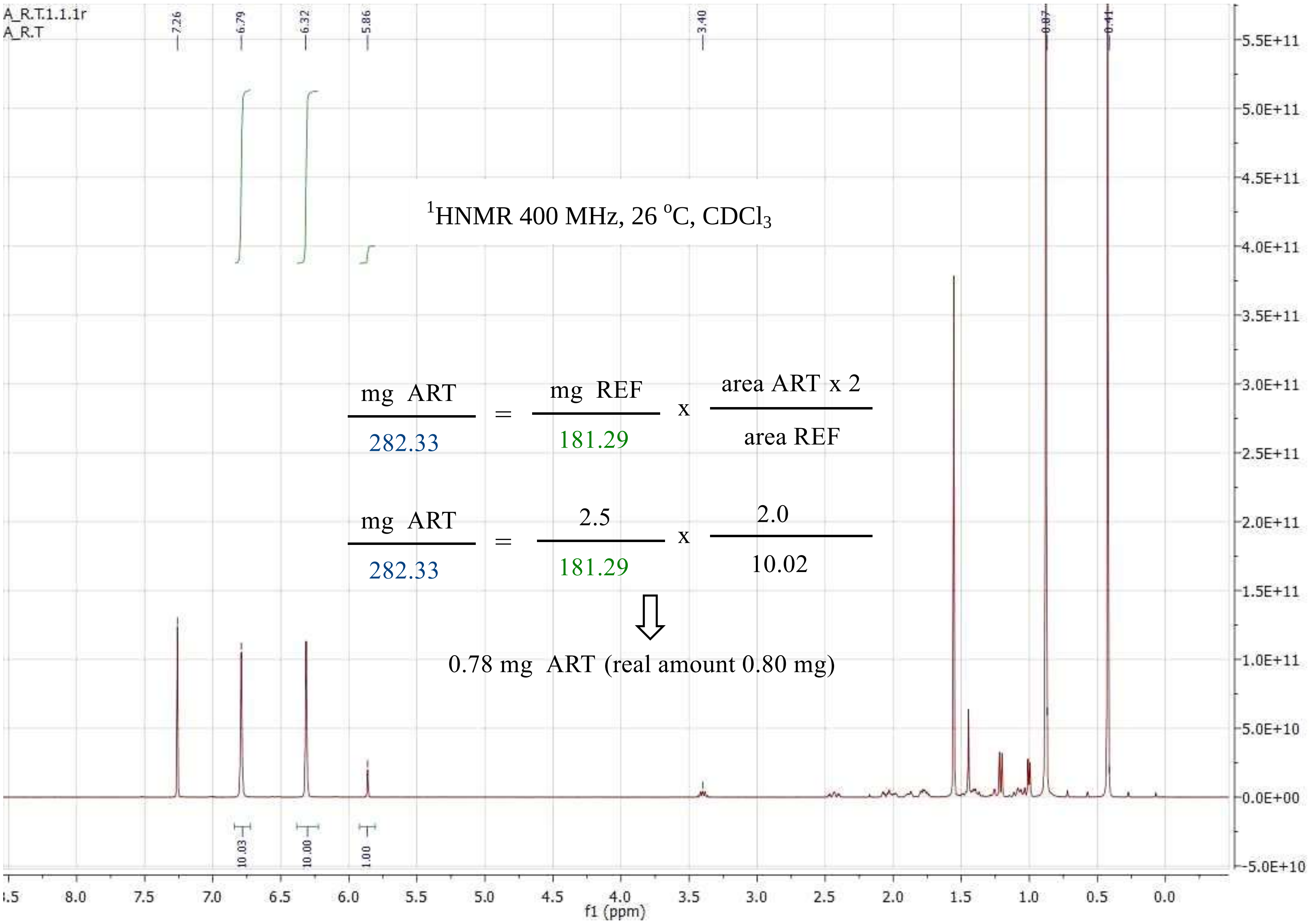

Supplementary Figure 8. First validations: mixtures of reference and artemisinin-method II. ^{1}H NMR spectra 3.

Second validations: Mixtures of references and artemisinin in a model plant extract – Method II

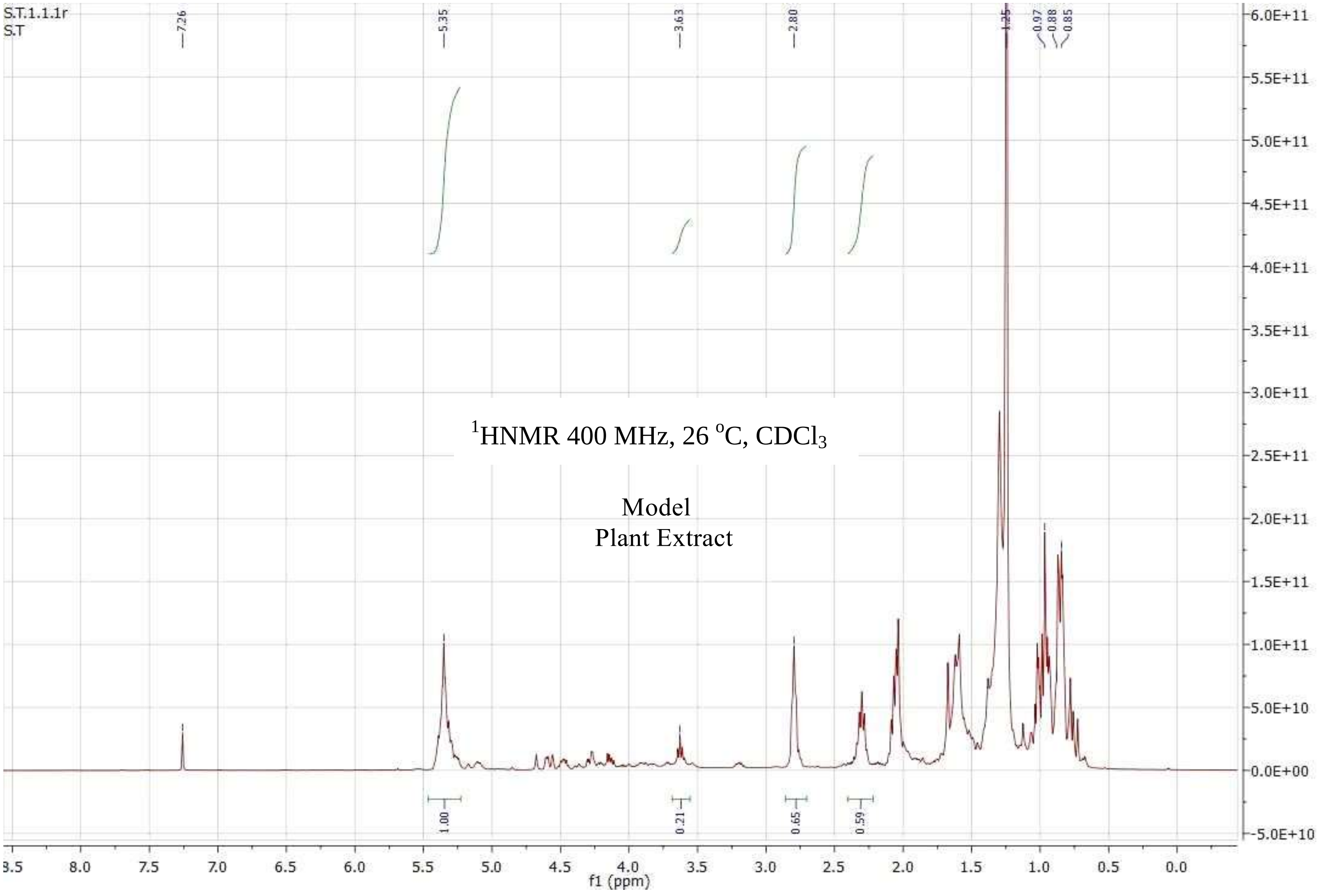

Supplementary Figure 9. Second validations: Mixtures of references and artemisinin in a model plant extract – Method II. ^{1}H NMR spectra of a model plant extract.

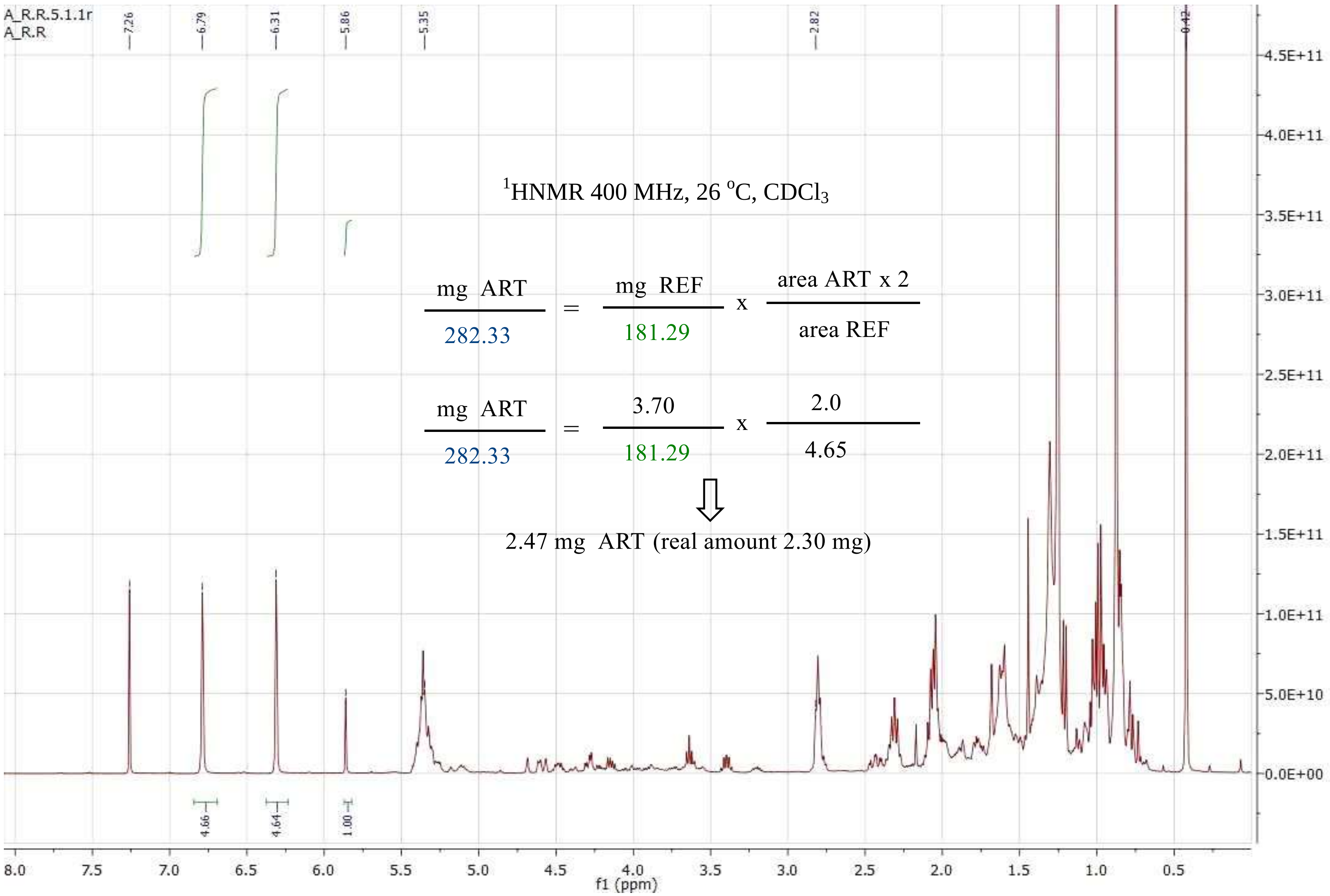

Supplementary Figure 10. Second validations: mixtures of reference and artemisinin in a model plant extract-method II. ^{1}H NMR spectra 1.

Third validations: mixtures of reference and *Artemisia annua* extract-method I

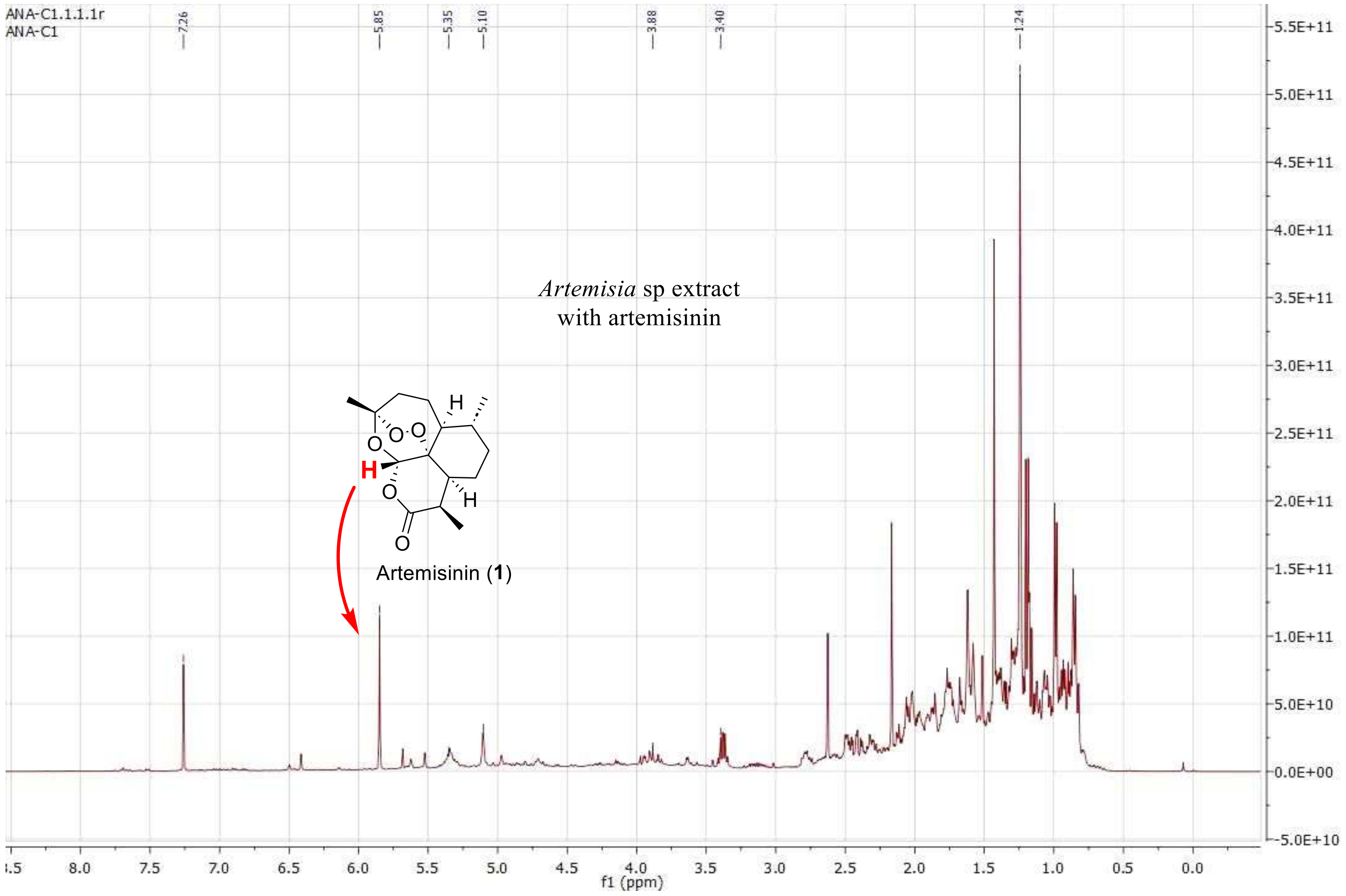

Supplementary Figure 11. Third validations: mixtures of reference and *A. annua* extract-method I. ^{1}H NMR spectra of *Artemisia annua* plant extract.

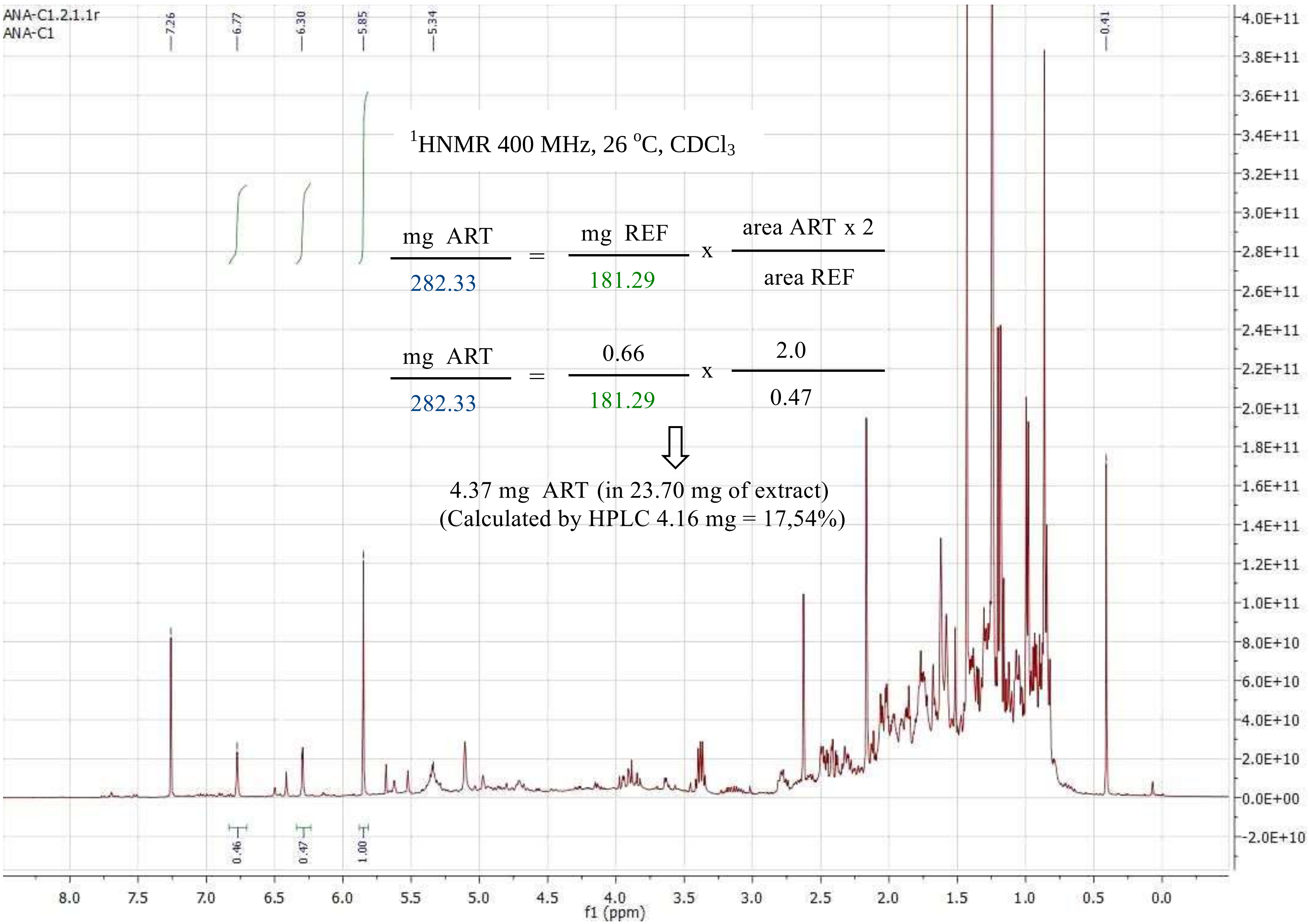

Supplementary Figure 12. Third validations: mixtures of reference and *A. annua* extract-method I. ^{1}H NMR spectra 1.

Third validations: mixtures of reference and *Artemisia annua* extract-method I

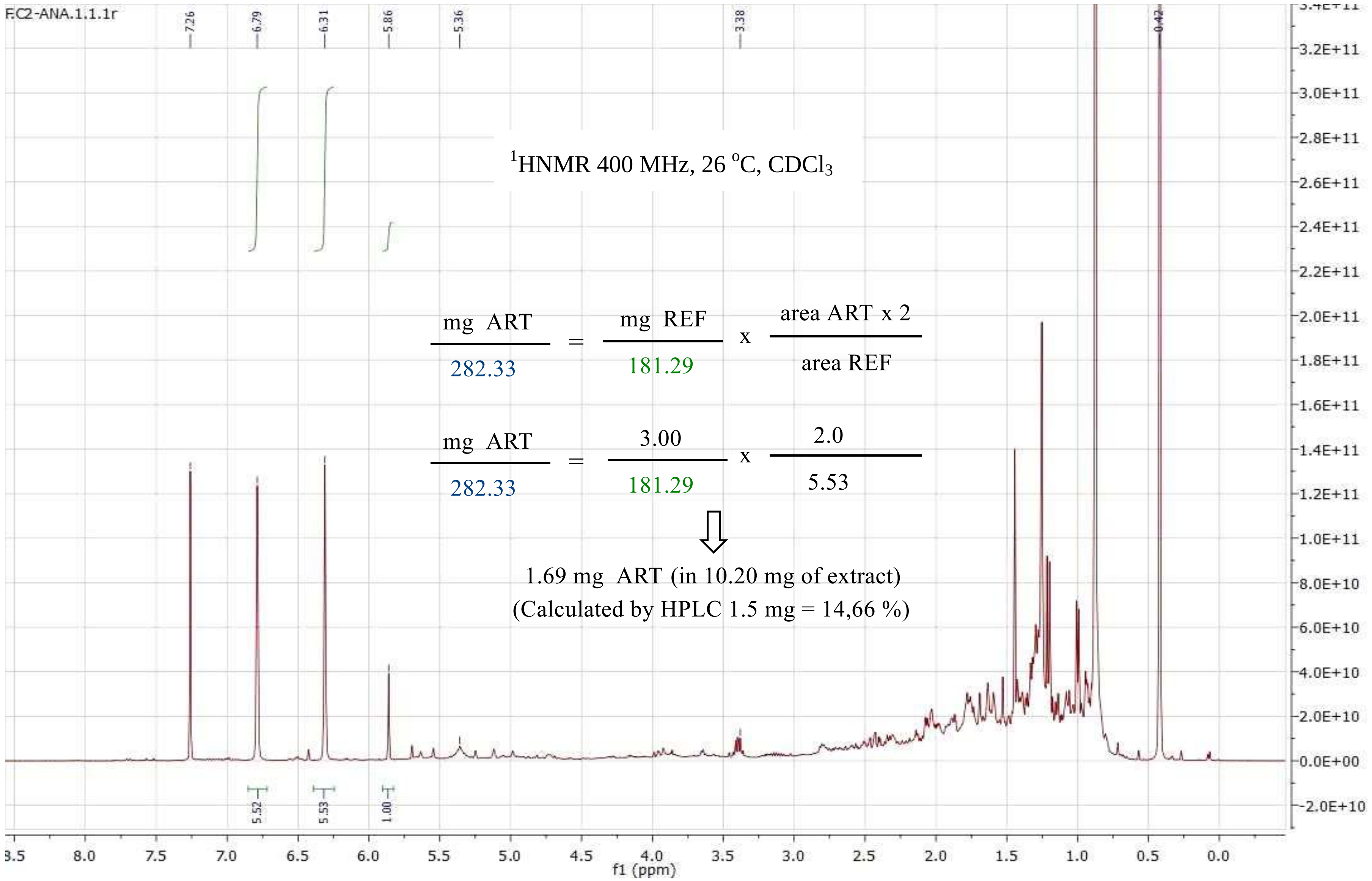

Supplementary Figure 13. Third validations: mixtures of reference and *A. annua* extract-method II. ^{1}H NMR spectra 1.

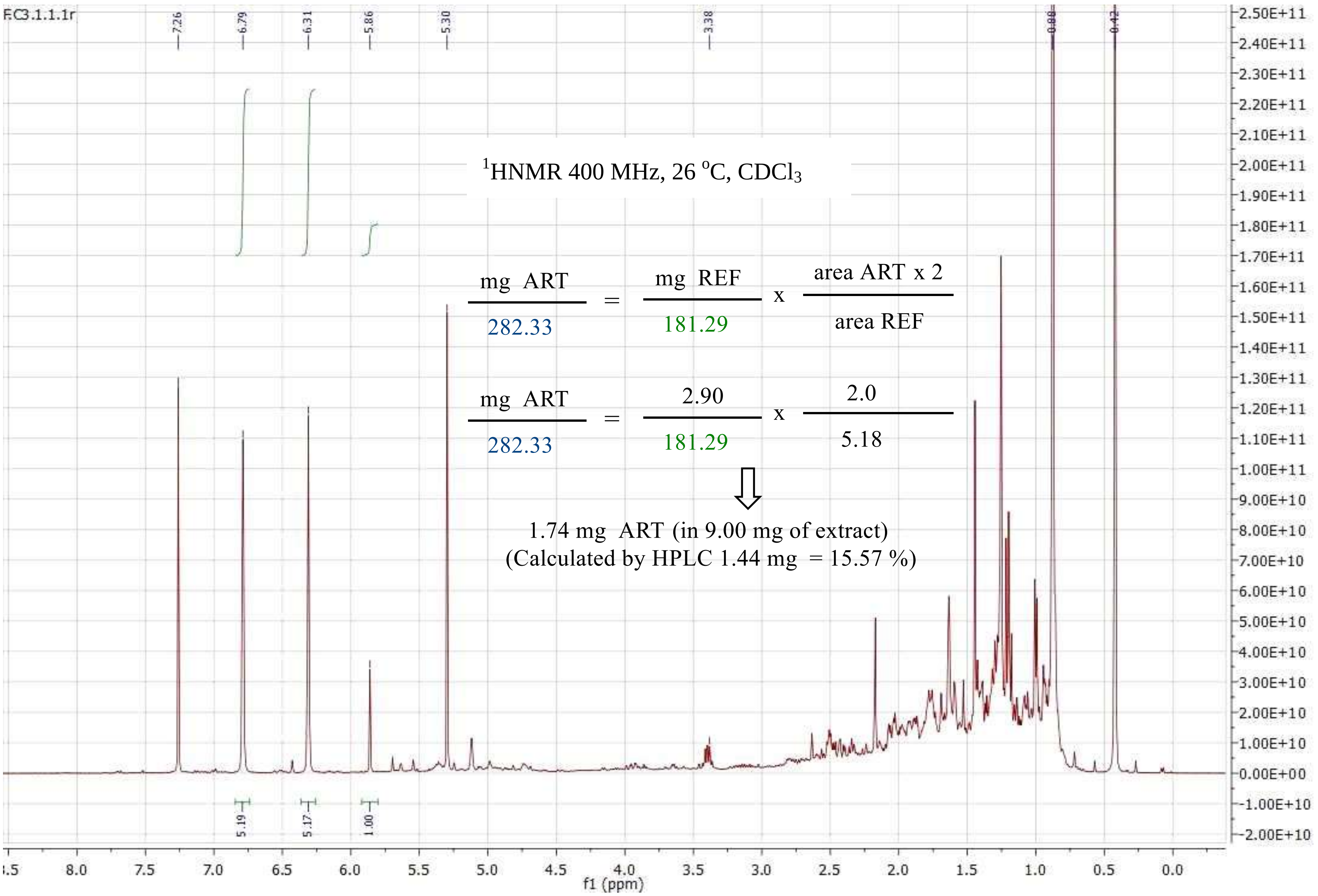

Supplementary Figure 14. Third validations: mixtures of reference and *A. annua* extract-method II. ^{1}H NMR spectra 2.

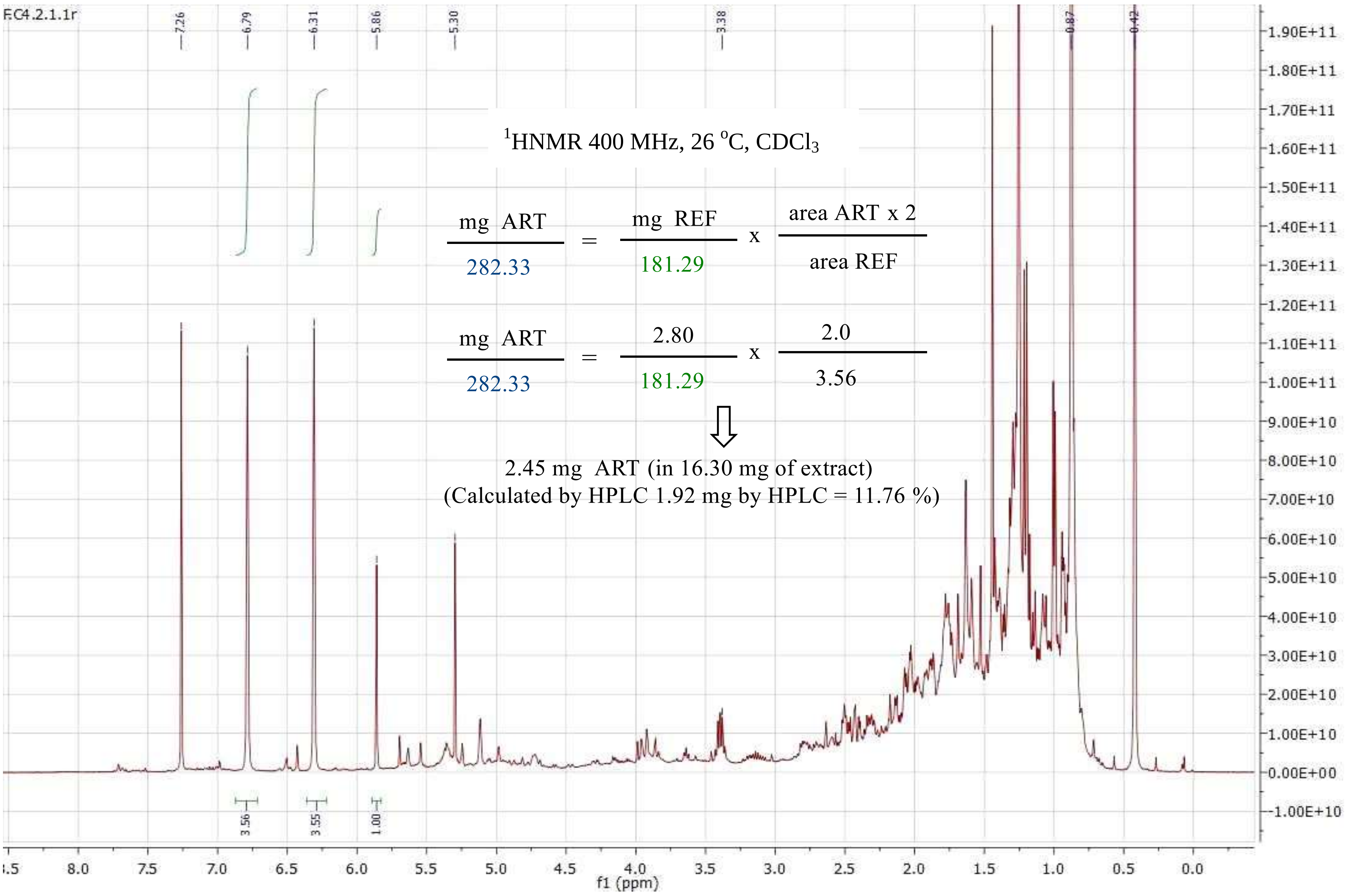

Supplementary Figure 15. Third validations: mixtures of reference and *A. annua* extract-method II. ^{1}H NMR spectra 3.

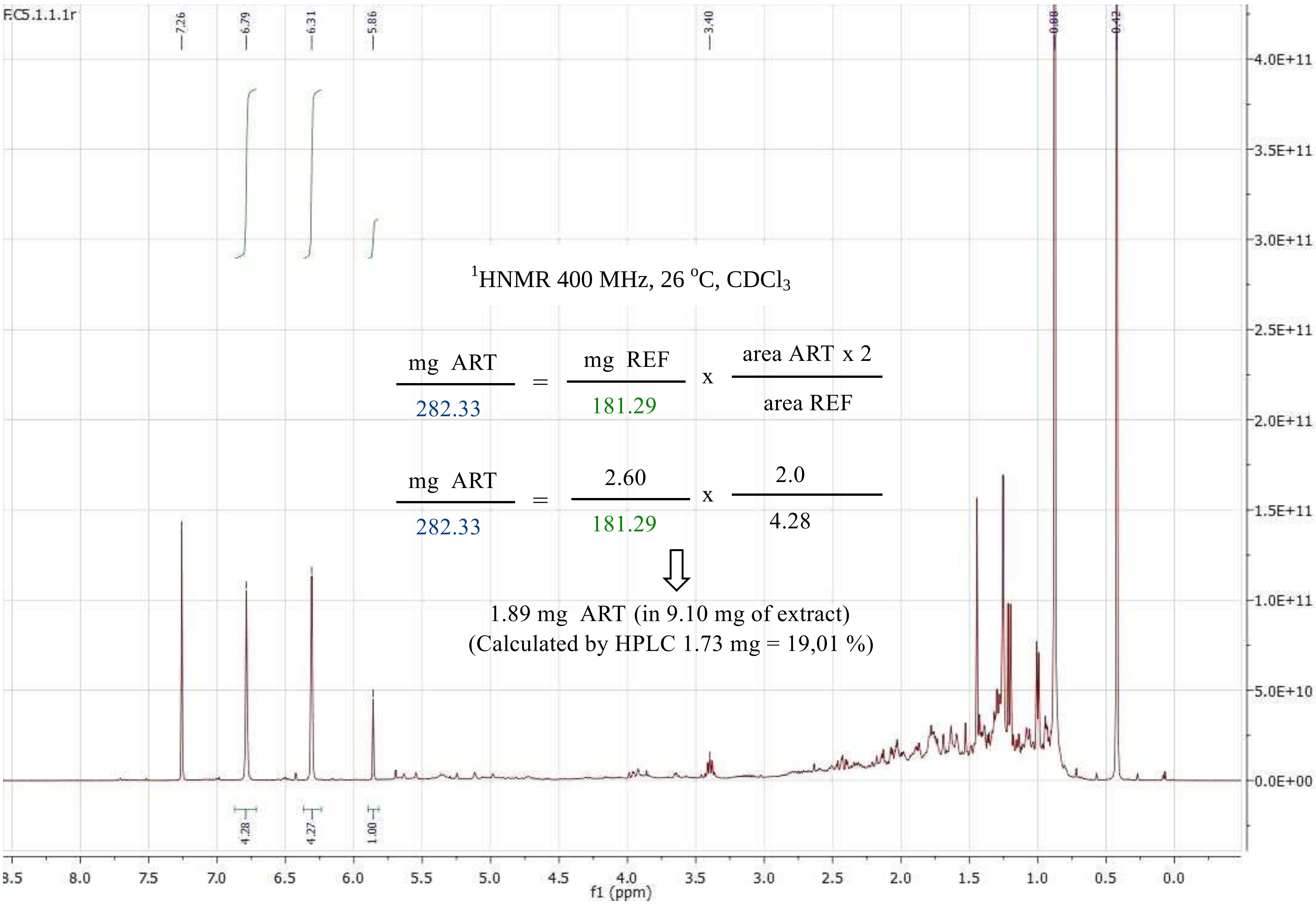

Supplementary Figure 16. Third validations: mixtures of reference and *A. annua* extract-method II. ^{1}H NMR spectra 4.

Validation of Method II to determine other natural products in a model plant extract

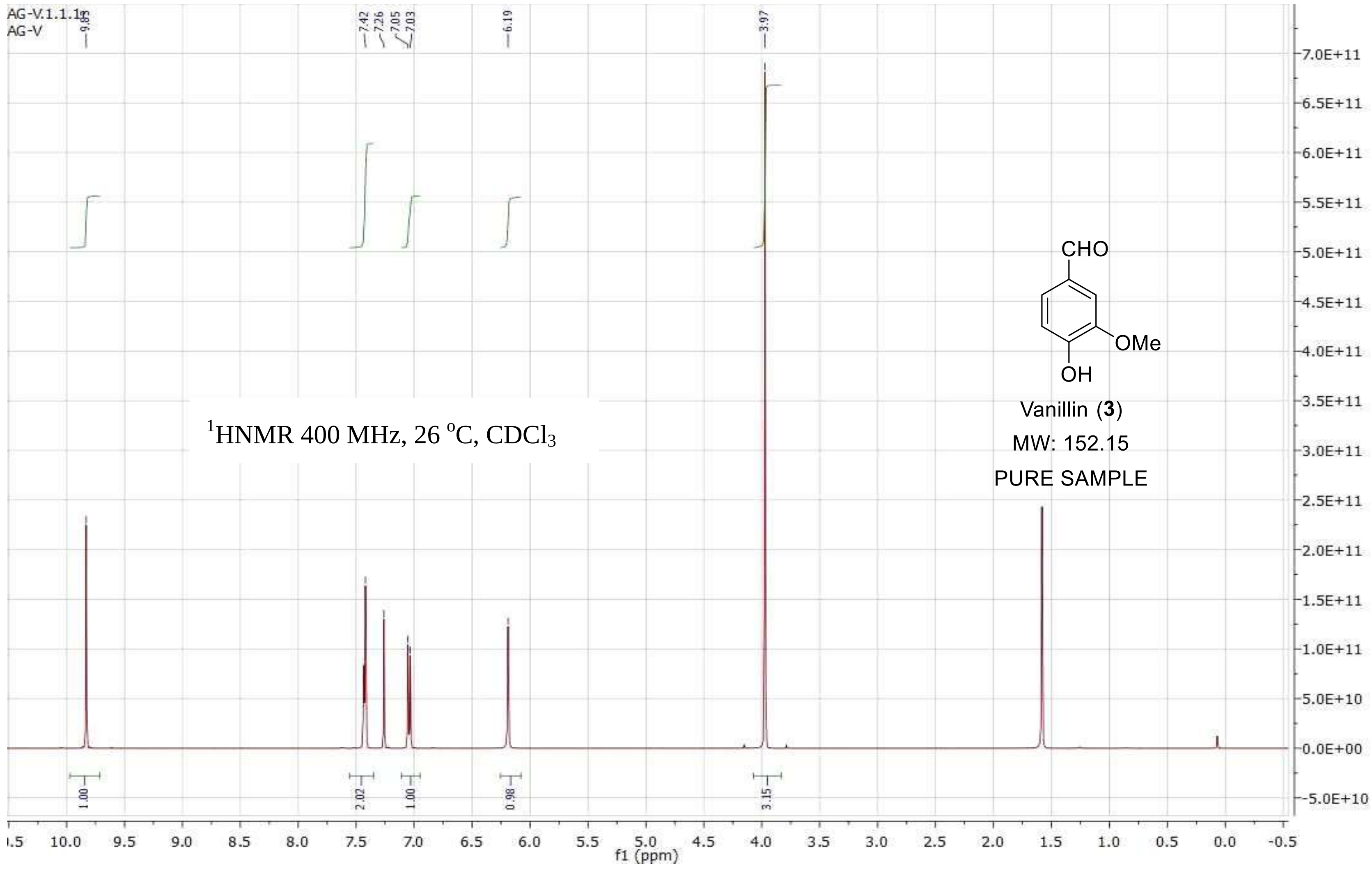

Supplementary Figure 17. Validation of method II to determine other natural products in a model plant extract. ^{1}H NMR spectra of vanillin (**3**).

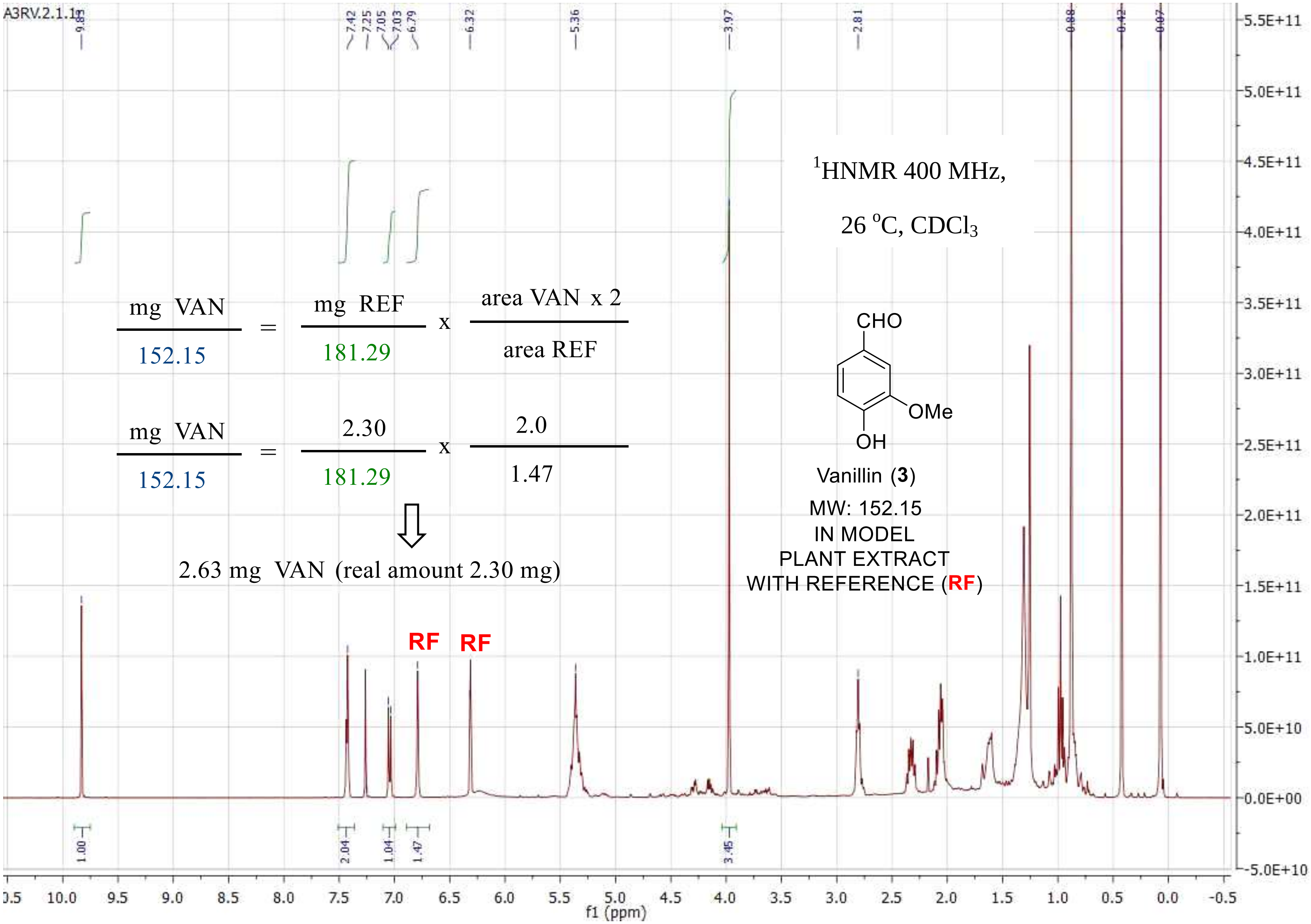

Supplementary Figure 18. Validation of method II to determine other natural products in a model plant extract. ^{1}H NMR spectra of vanillin (**3**) in a model plant extract with the reference.

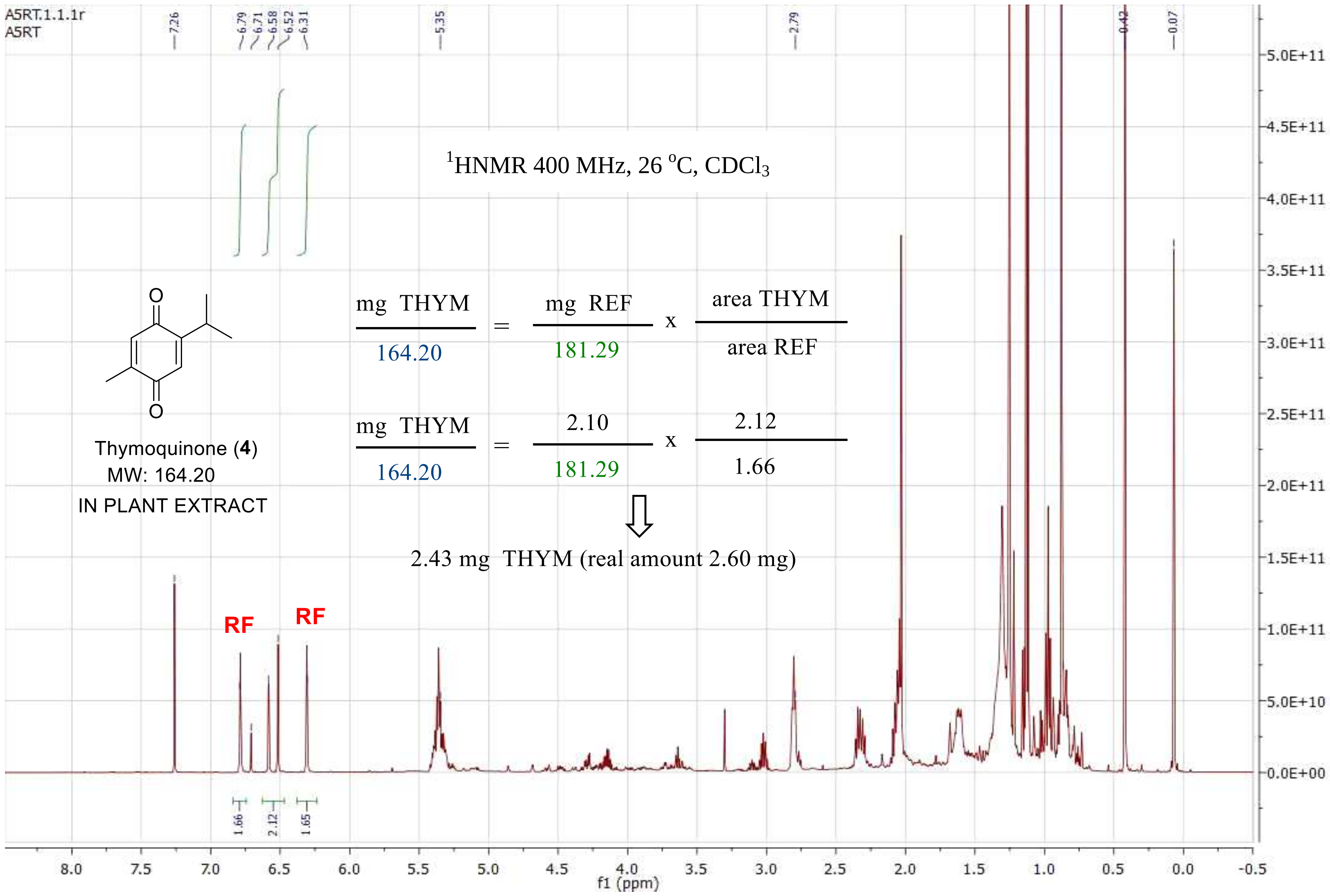

Supplementary Figure 19. Validation of method II to determine other natural products in a model plant extract. ^{1}H NMR spectra of thymoquinone (**4**) in a model plant extract with the reference.

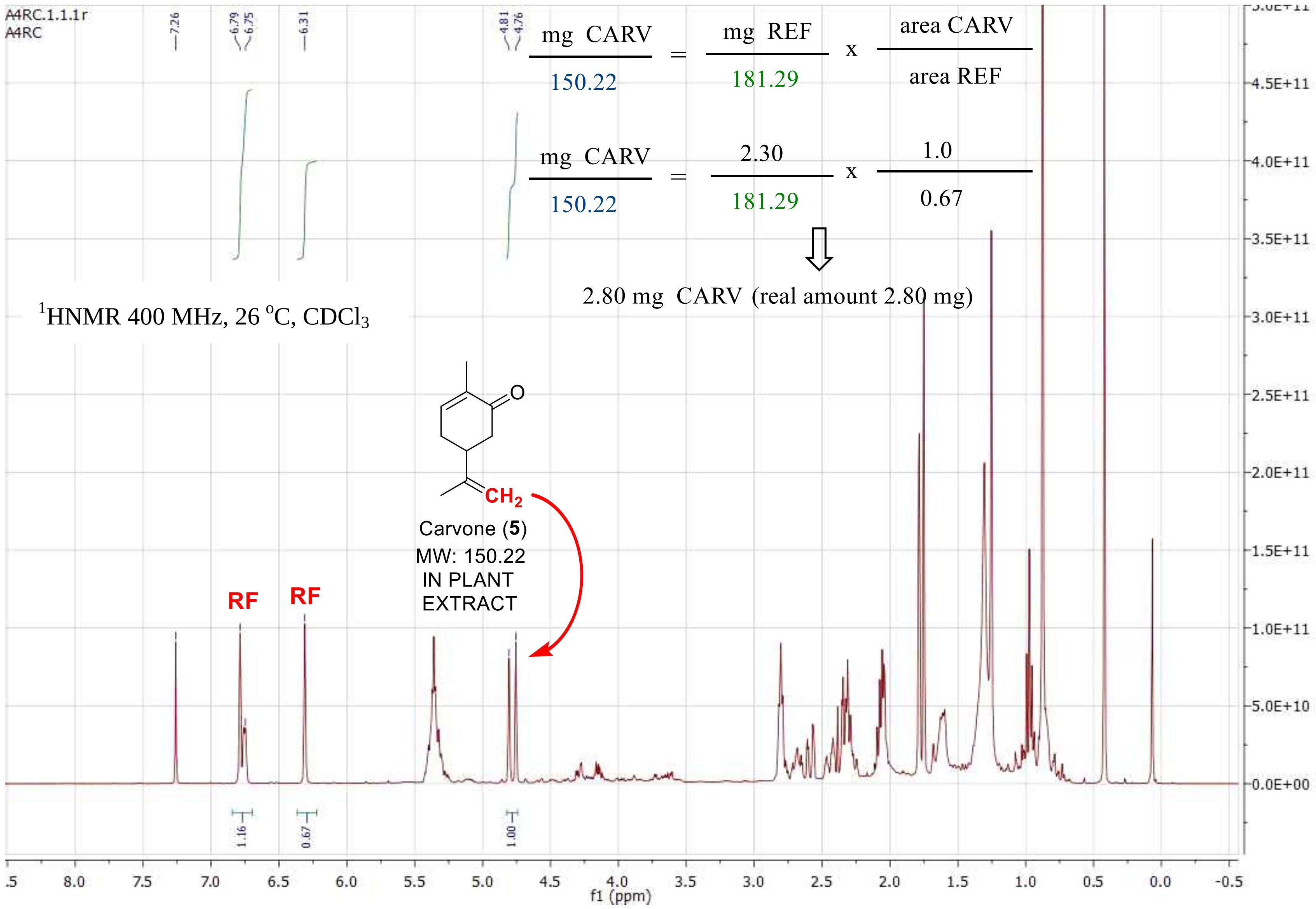

Supplementary Figure 20. Validation of method II to determine other natural products in a model plant extract. ^{1}H NMR spectra of carvone (**5**) in a model plant extract with the reference.

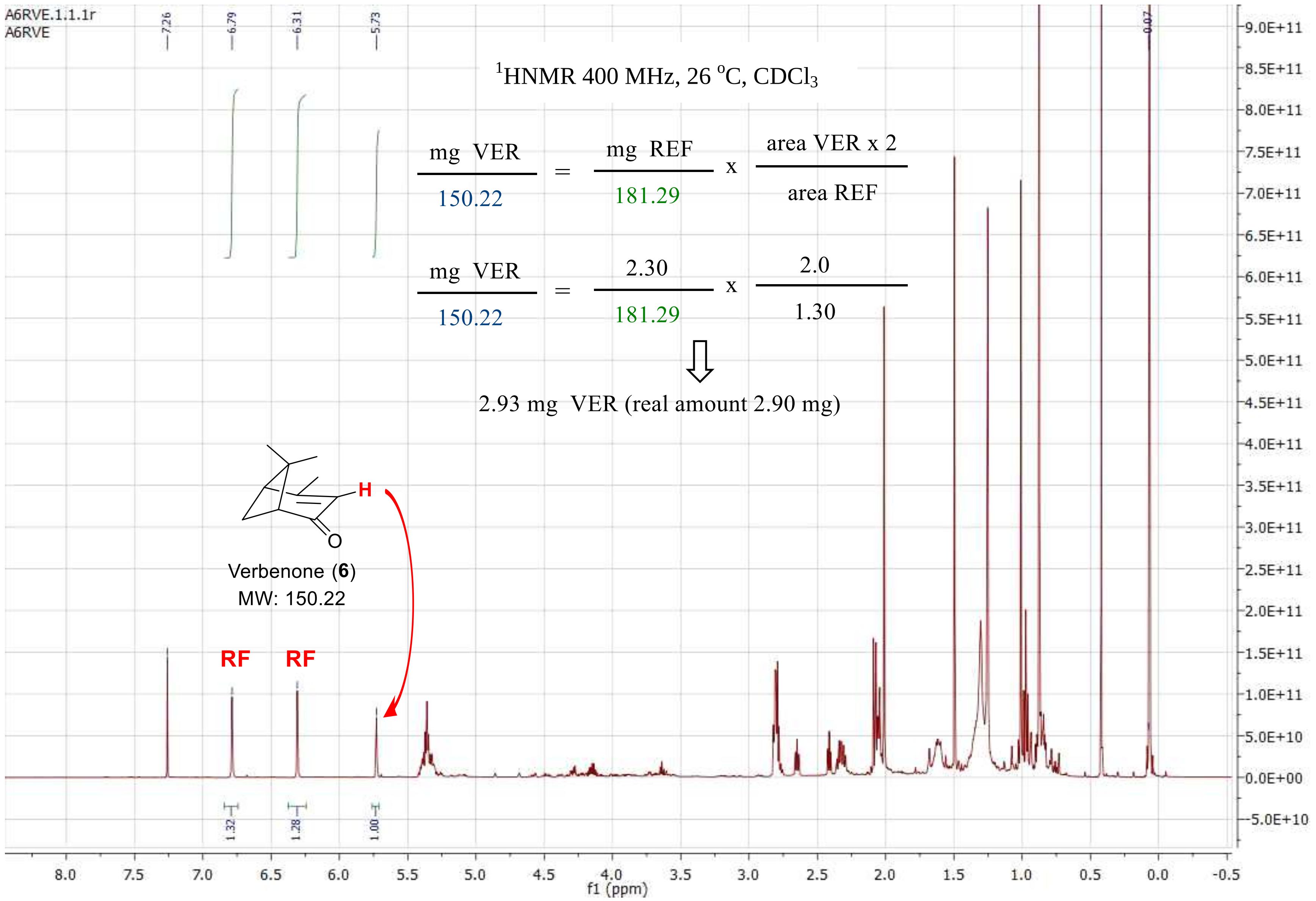

Supplementary Figure 21. Validation of method II to determine other natural products in a model plant extract. ^{1}H NMR spectra of verbenone (**6**) in a model plant extract with the reference.

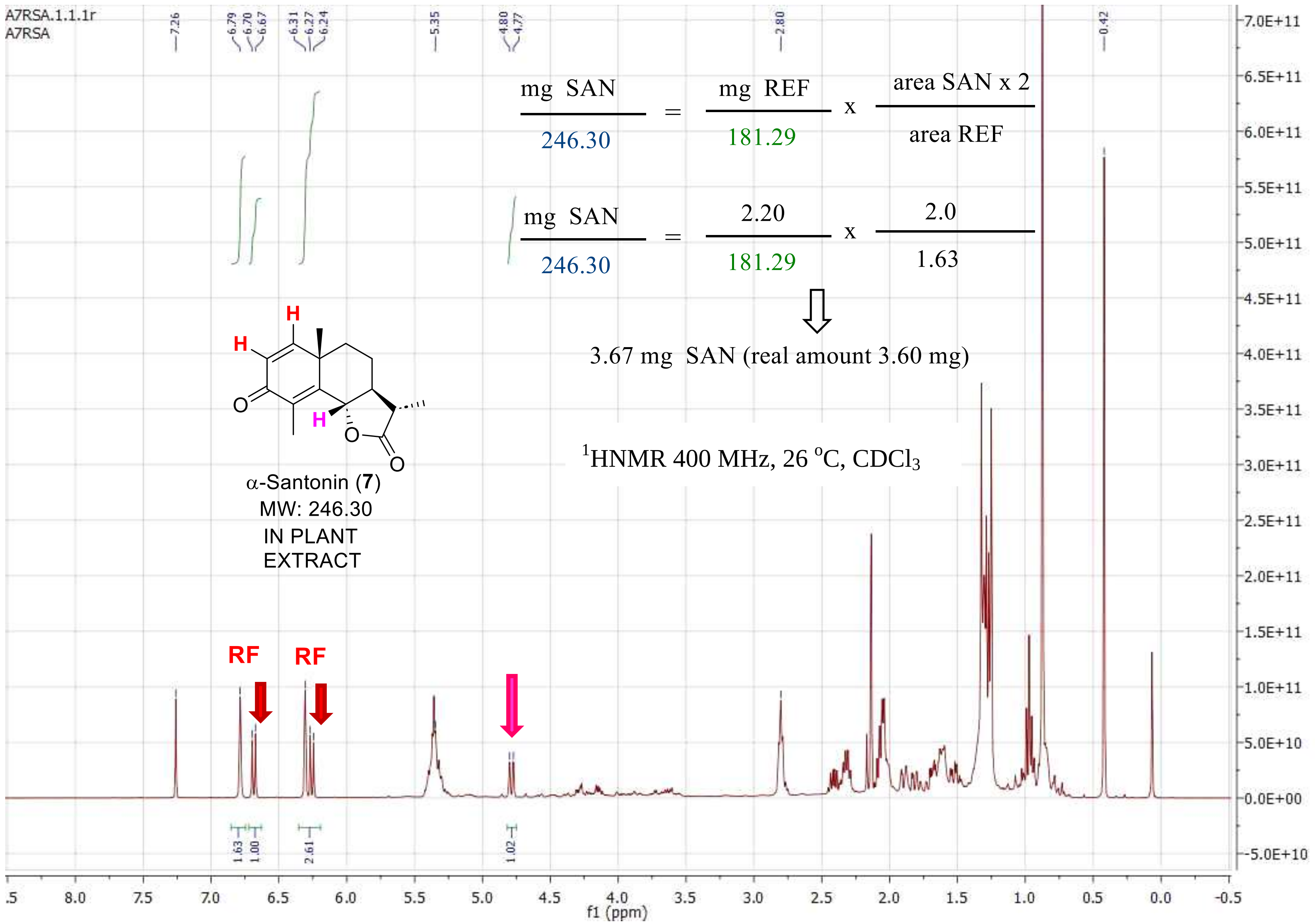

Supplementary Figure 22. Validation of method II to determine other natural products in a model plant extract. ^{1}H NMR spectra of α-santonin (**7**) in a model plant extract with the reference.

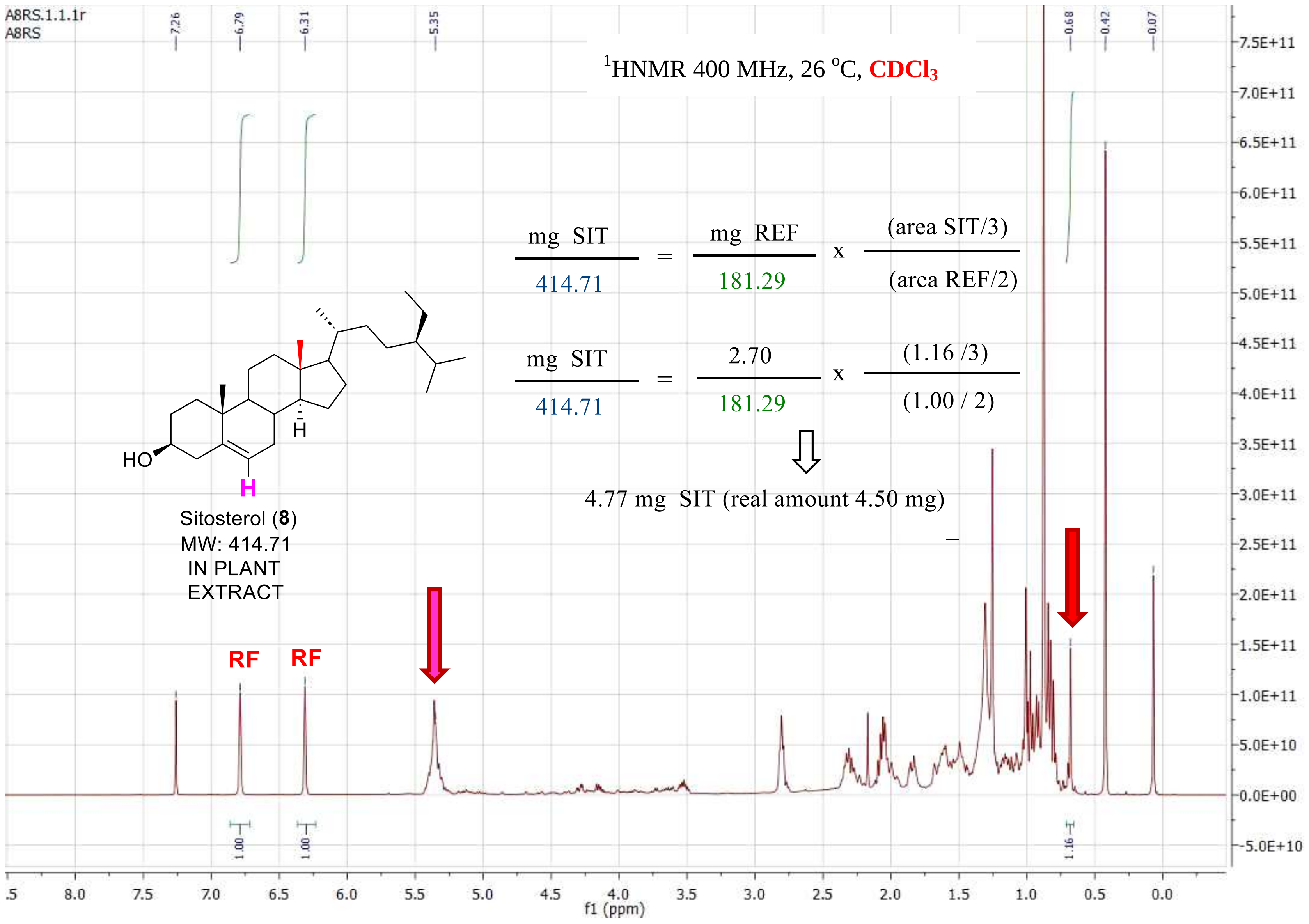

Supplementary Figure 23. Validation of method II to determine other natural products in a model plant extract. ^{1}H NMR spectra of sitosterol (**8**) in a model plant extract with the reference in $CDCl_3$ as deuterated solvent.

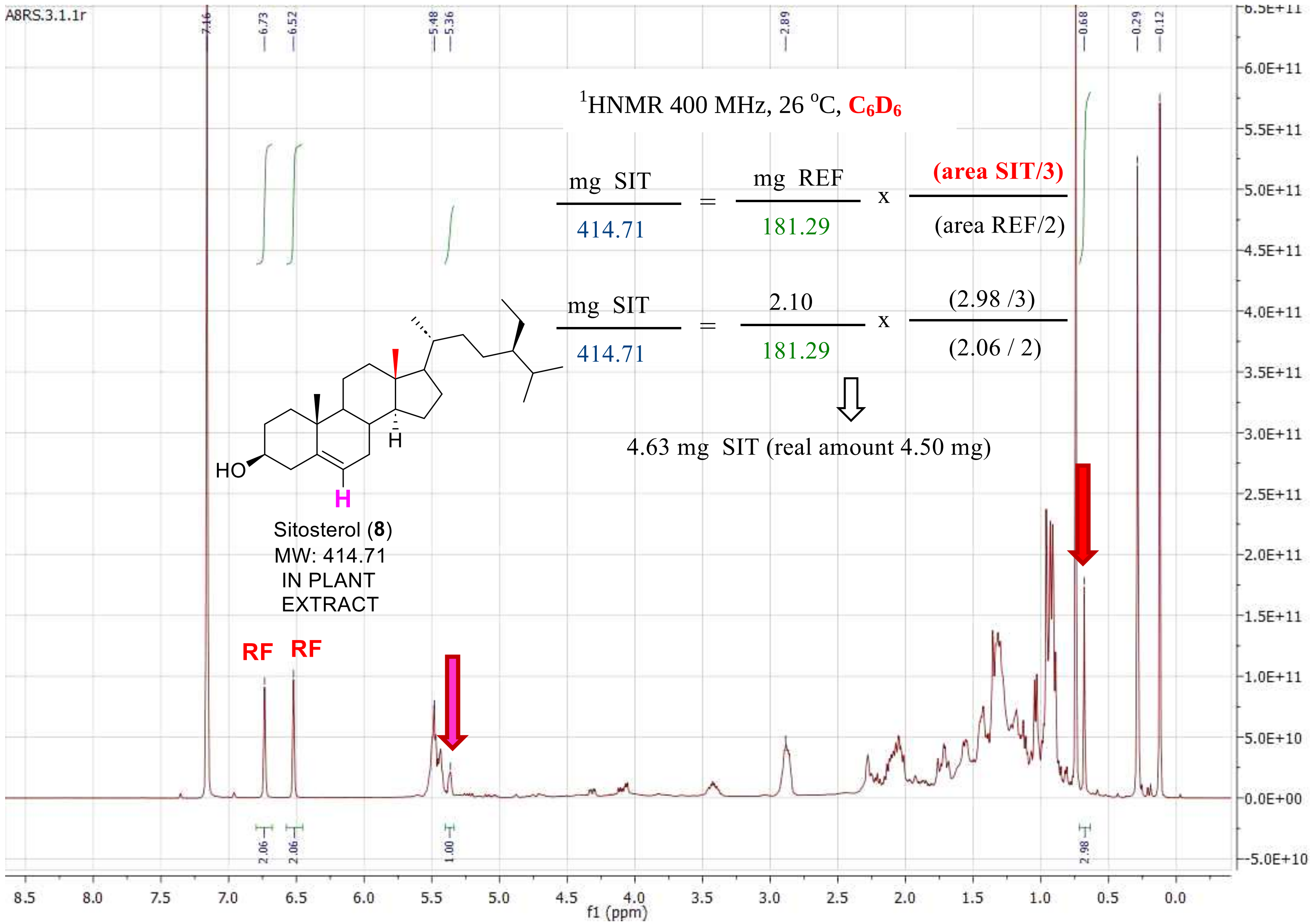

Supplementary Figure 24. Validation of method II to determine other natural products in a model plant extract. ^{1}H NMR spectra of sitosterol (**8**) in a model plant extract with the reference in C_6D_6 as deuterated solvent.

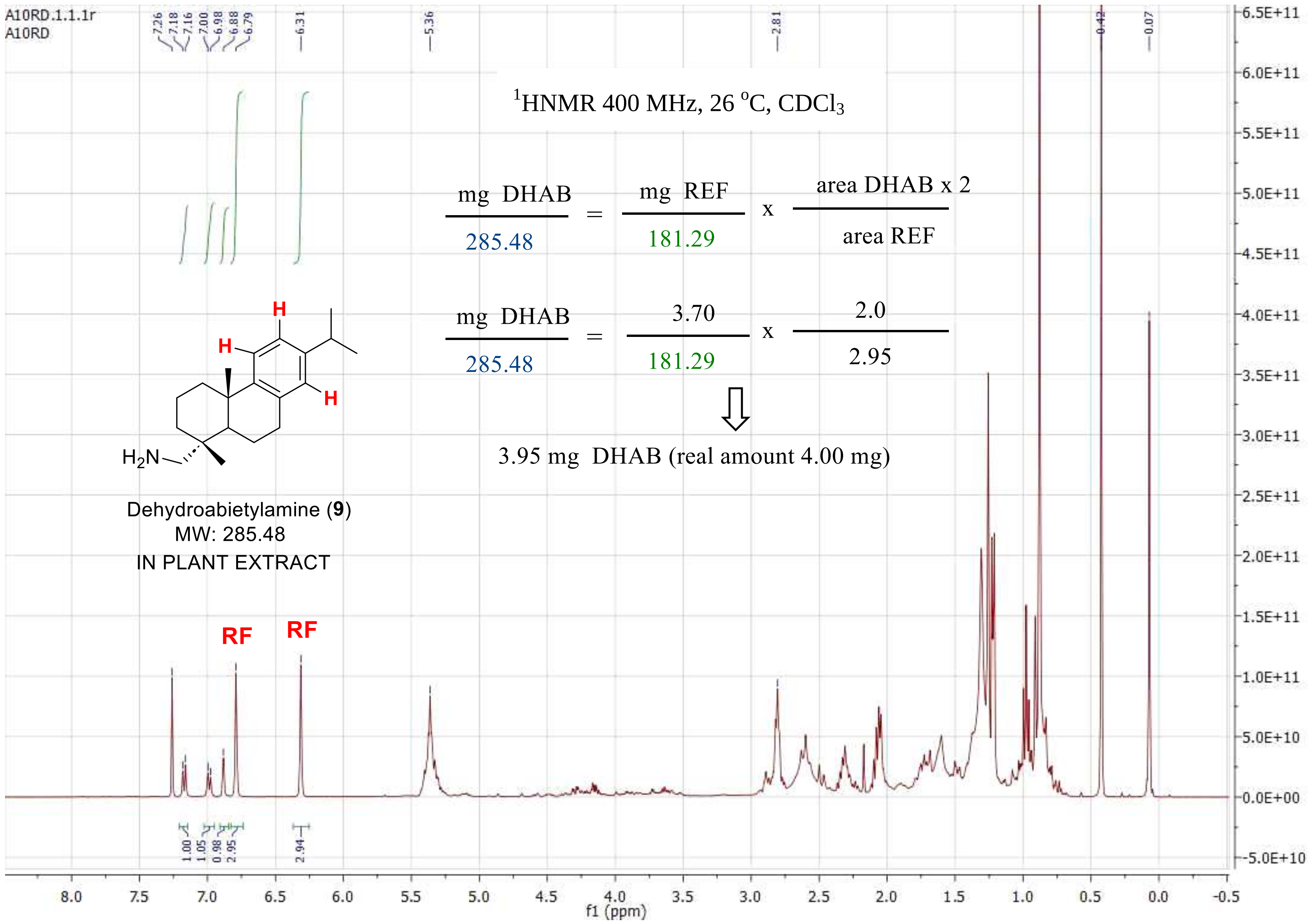

Supplementary Figure 25. Validation of method II to determine other natural products in a model plant extract. ^{1}H NMR spectra of dehydroabietylamine (**9**) in a model plant extract.

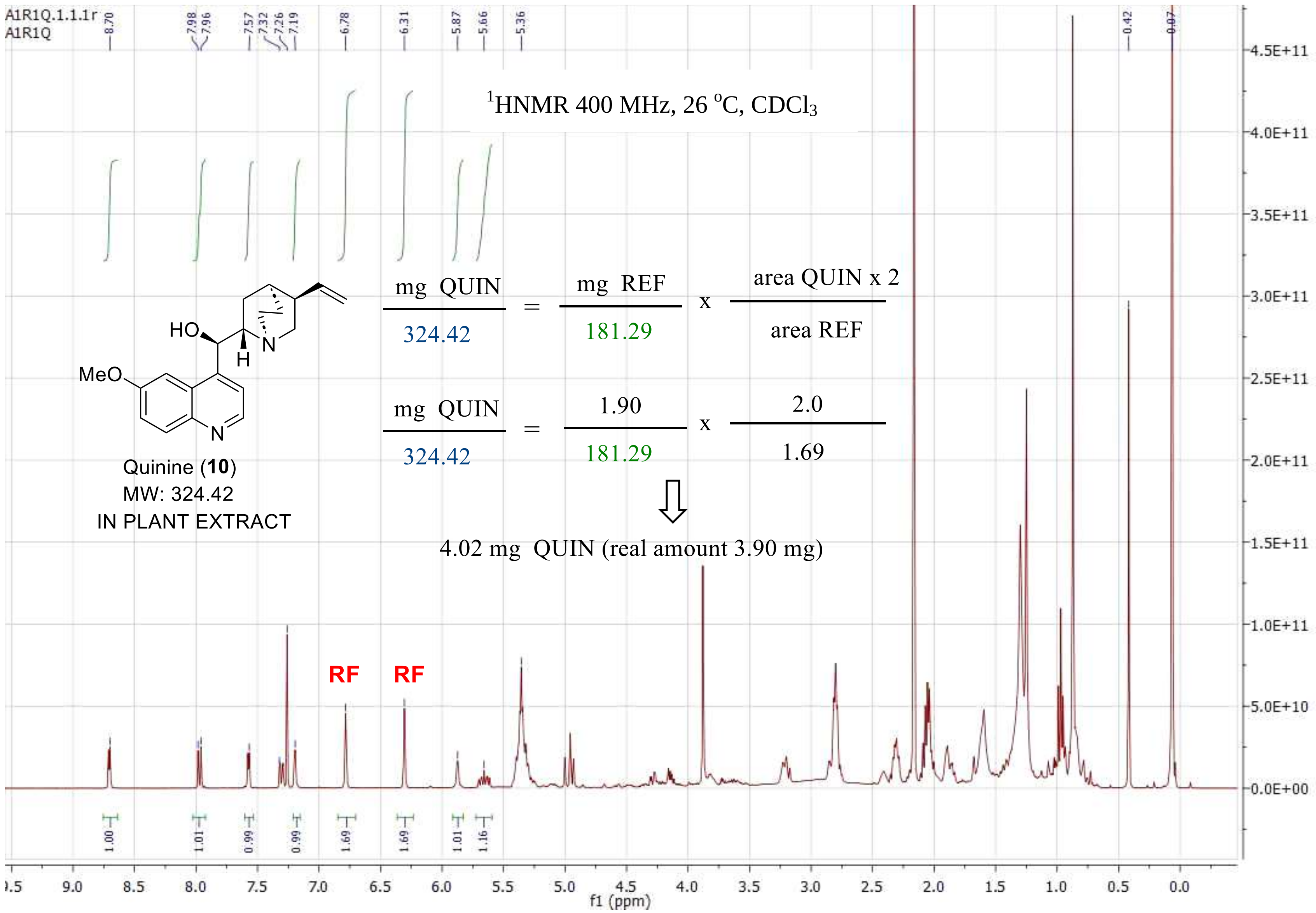

Supplementary Figure 26. Validation of method II to determine other natural products in a model plant extract. ^{1}H NMR spectra of quinine (**10**) in a model plant extract.

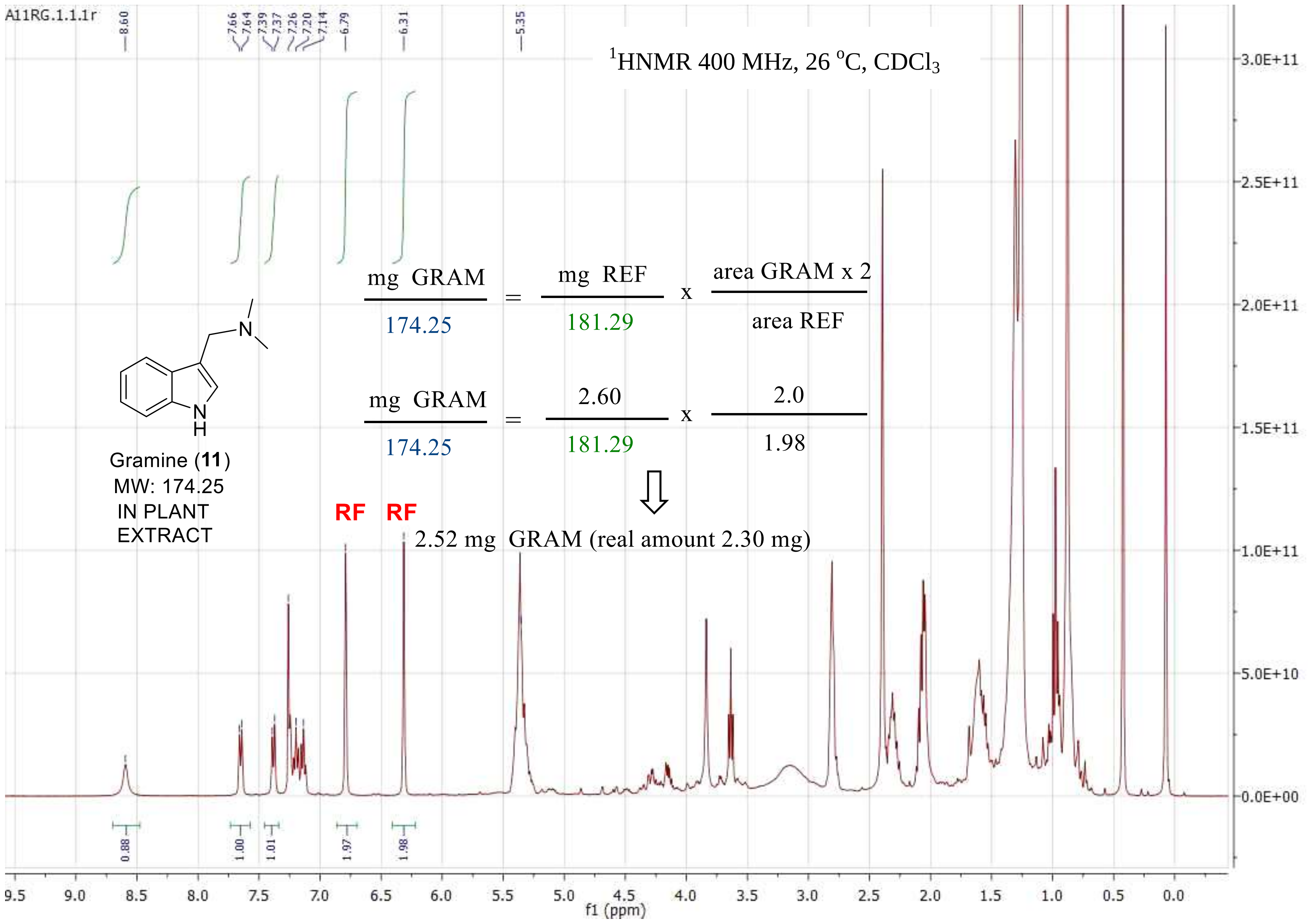

Supplementary Figure 27. Validation of method II to determine other natural products in a model plant extract. ^{1}H NMR spectra of gramine (**11**) in a model plant extract.
